# Endothelial Cells Support Persistent Gammaherpesvirus 68 Infection

**DOI:** 10.1371/journal.ppat.1000152

**Published:** 2008-09-12

**Authors:** Andrea Luísa Suárez, Linda Faye van Dyk

**Affiliations:** 1 Department of Microbiology and Program in Molecular Biology, University of Colorado Denver School of Medicine, Aurora, Colorado, United States of America; 2 Department of Immunology, University of Colorado Denver School of Medicine, Aurora, Colorado, United States of America; University of Southern California School of Medicine, United States of America

## Abstract

A variety of human diseases are associated with gammaherpesviruses, including neoplasms of lymphocytes (e.g. Burkitt's lymphoma) and endothelial cells (e.g. Kaposi's sarcoma). Gammaherpesvirus infections usually result in either a productive lytic infection, characterized by expression of all viral genes and rapid cell lysis, or latent infection, characterized by limited viral gene expression and no cell lysis. Here, we report characterization of endothelial cell infection with murine gammaherpesvirus 68 (γHV68), a virus phylogenetically related and biologically similar to the human gammaherpesviruses. Endothelial cells supported γHV68 replication *in vitro*, but were unique in that a significant proportion of the cells escaped lysis, proliferated, and remained viable in culture for an extended time. Upon infection, endothelial cells became non-adherent and altered in size, complexity, and cell-surface protein expression. These cells were uniformly infected and expressed the lytic transcription program based on detection of abundant viral gene transcripts, GFP fluorescence from the viral genome, and viral surface protein expression. Additionally, endothelial cells continued to produce new infectious virions as late as 30 days post-infection. The outcome of this long-term infection was promoted by the γHV68 v-cyclin, because in the absence of the v-cyclin, viability was significantly reduced following infection. Importantly, infected primary endothelial cells also demonstrated increased viability relative to infected primary fibroblasts, and this increased viability was dependent on the v-cyclin. Finally, we provide evidence for infection of endothelial cells *in vivo* in immune-deficient mice. The extended viability and virus production of infected endothelial cells indicated that endothelial cells provided a source of prolonged virus production and identify a cell-type specific adaptation of gammaherpesvirus replication. While infected endothelial cells would likely be cleared in a healthy individual, persistently infected endothelial cells could provide a source of continued virus replication in immune-compromised individuals, a context in which gammaherpesvirus-associated pathology frequently occurs.

## Author Summary

Various human diseases are associated with gammaherpesvirus infections, including neoplasms of lymphocytes (e.g. Burkitt's lymphoma) and endothelial cells (e.g. Kaposi's sarcoma). Gammaherpesvirus infection of cells usually results in either productive infection that is characterized by new virus production and rapid destruction of the host cell, or latent infection that is characterized by long-term carriage of viral DNA in intact cells that do not produce virus. Here, we characterize endothelial cell infection using a small animal model of gammaherpesvirus infection and disease. While infection of endothelial cells resulted in virus production as most cells do, infection of this cell type was unique in that cells remained intact and continued to proliferate. These intact, infected endothelial cells were significantly altered in appearance and gene expression compared to uninfected cells, changes predicted to impact endothelial cell growth and function. Endothelial cells were unique in their ability to support this type of persistent infection. These data demonstrate an additional mechanism, beyond latency, by which gammaherpesviruses may achieve long-term propagation, particularly in conditions of immune suppression. Our results suggest persistently infected cells as a therapeutic target for prevention/treatment of chronic disease, and may provide a mouse model for future testing.

## Introduction

Endothelial cells create a physical barrier on the luminal surface of blood and lymphatic vessels. This barrier must be traversed by blood-borne pathogens and immune cells trafficking between tissues and the bloodstream. Many herpesviruses require systemic spread for persistence within a host, and therefore must cross such an endothelial cell barrier. To date, herpesviruses have been implicated as potential initiators of arterial injury, endothelial dysfunction, and local inflammation, possibly contributing to the pathogenesis of atherosclerosis [1]–[4]. Human cytomegalovirus (HCMV), a betaherpesvirus, infects endothelial cells *in vivo*. Studies have shown that infected endothelial cells play a role in HCMV dissemination and pathogenesis [5]. Endothelial cells exhibit regional specialization in gene expression and morphology depending on the local physiologic demands of their respective organs and tissues [6]. In light of this diversity, it is not surprising that endothelial cells from different tissues differ in their susceptibility to HCMV infection [7]. Kaposi's sarcoma-associated herpesvirus (KSHV), a human gammaherpesvirus, also infects endothelial cells *in vivo*. More importantly, KSHV is the causative agent of the endothelial cell neoplasm, Kaposi's sarcoma (KS). The murine gammaherpesvirus, γHV68, has been detected in aortic endothelium after infection of apoE deficient mice, as well as on the luminal surface of explanted aortas infected *in vitro* [8]. While a relationship between gammaherpesviruses and endothelial cells has been noted, the role of endothelial cells in chronic gammaherpesvirus infection and pathogenesis is ill-defined.

Gammaherpesviruses are a lymphotropic family of viruses associated with a broad spectrum of malignancies and lymphoproliferative diseases. These oncogenic viruses persist for the life of the host by establishing and maintaining a latent infection. Because gammaherpesviruses are extremely host-specific, studying the human viruses Epstein-Barr virus (EBV) and KSHV in non-human systems does not mimic natural infection. γHV68 is a natural pathogen of wild murid rodents and therefore provides a valuable small animal model of gammaherpesvirus infection [9]–[13]. This virus has important biological and genetic similarities to the human gammaherpesviruses, and results in a variety of pathologies in defined mouse models. Primary γHV68 infection is characterized by virus replication in lung epithelial cells, and the establishment of latency in B cells, dendritic cells, and macrophages [12]–[16]. Persistent γHV68 infection also occurs in lung epithelial cells [17]. The role of endothelial cells in γHV68 infection has not been explored to date.

At the cellular level, gammaherpesvirus infection encompasses two broadly defined outcomes: productive, lytic replication and non-productive, latent infection. During lytic replication, viral DNA is amplified and host cell machinery is utilized for the production of viral progeny. Viral genes are actively transcribed and translated, contributing to new virus production. Ultimately, the cell is lysed and infectious virus released. In light of the fact that this process occurs quickly (24 to 48 hours *in vitro*) our understanding of virus-host interactions during primary infection is quite limited. During latency, viral DNA is not amplified, but instead is maintained as a nuclear episome in latently infected cells, and little viral gene transcription occurs. Latently infected cells remain intact and do not produce new infectious virus. Furthermore, it is thought that these long-lived latent cells may serve as the major mechanism by which gammaherpesviruses promote life-long infection of their hosts.

Here we characterize γHV68 infection of endothelial cells. To date, *in vitro* infection of most cells with γHV68 supports significant viral replication and results in complete cell lysis (data not shown, van Dyk). We have infected both primary endothelial cells and endothelial cell lines and demonstrated that they produce comparable amounts of virus as fibroblasts. However, whereas fibroblasts were mostly lysed by 36–48 hours post-infection, a significant percentage of infected endothelial cells remained intact. Analysis of endothelial cell lines revealed that these intact cells 1) were actively infected and undergoing the lytic transcriptional program, 2) continued to proliferate for a prolonged time after infection, 3) were altered in morphology and cell-surface protein expression, compared to uninfected endothelial cells, and 4) continued to release infectious virions as far as 30 days post-infection. In the absence of the γHV68 viral cyclin, endothelial cell viability was significantly decreased after infection, indicating that an active viral process was responsible for promoting the outcome of infection in endothelial cells. Of major significance, we also provided evidence of endothelial cell infection in primary cells and *in vivo* in immune-deficient mice. These data demonstrated that endothelial cells supported persistent, productive gammaherpesvirus infection, an outcome not previously reported for γHV68 or other gammaherpesviruses. Together, these data indicated the potential of the endothelial cell as a critical cell type in γHV68 pathogenesis, and open a new avenue of research into herpesvirus manipulation of endothelial cell biology.

## Results

### Endothelial cells supported γHV68 replication

**Figure 1:**
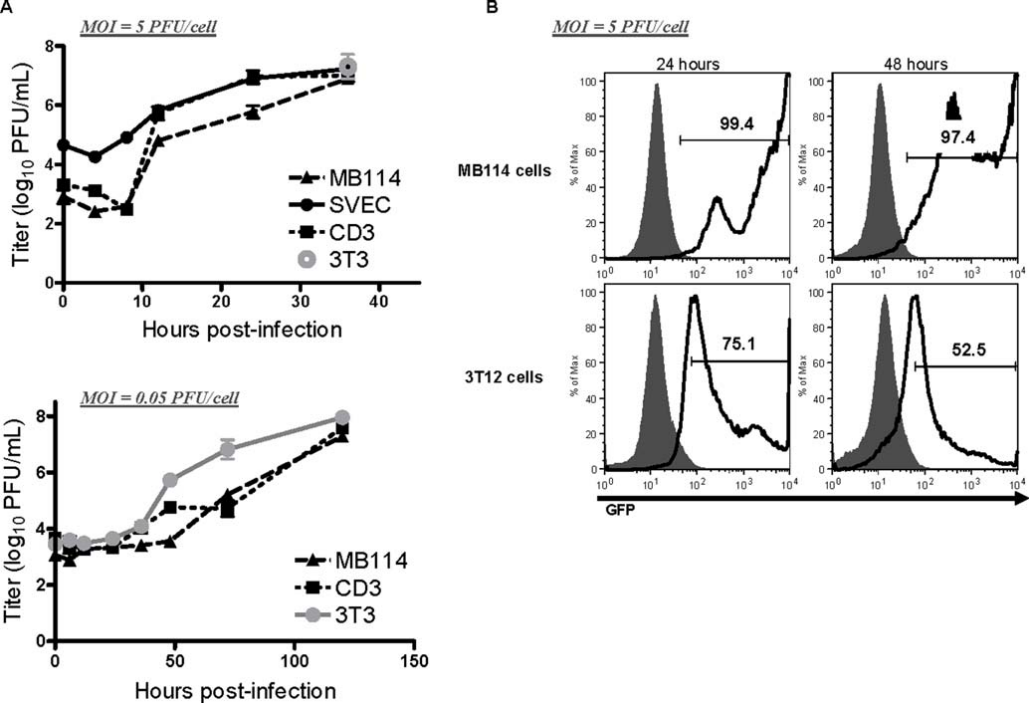
Endothelial cells supported γHV68 replication. *In vitro* growth assays of γHV68 in MB114, SVEC, and CD3 endothelial cells. Cell were infected at the indicated MOI with either wildtype γHV68 (A) or γHV68-GFP (B) and harvested at the indicated times. (A) Samples were multiply freeze-thawed prior to quantification by plaque assay on NIH 3T12 cells. Top panel, γHV68 titer 36 hours post-infection in 3T3 fibroblasts compared to MB114, SVEC, and CD3 endothelial cells. n = 2–3. (B) Cells infected with γHV68-GFP were analyzed by flow cytometry for GFP expression at 24 and 48 hours post-infection. Fluorescence was determined relative to cells infected with wildtype γHV68 (grey). Histograms are representative of two independent infections.

Endothelial cells are heterogeneous in nature, express unique surface antigens, and differ in their susceptibility and response to infection by various pathogens [6]. In light of this diversity, we investigated endothelial cell lines from various anatomic locations, and from BALB/c and C57BL/6 mice, for their ability to support γHV68 replication. We analyzed the outcome of γHV68 infection in endothelial cell lines from lung (CD3), brain capillary (MB114), and lymph node (SVEC) by single-step and multi-step growth assays. Single step growth proceeded with kinetics similar to previously published infection of the fibroblast cell line, NIH 3T12 cells [18] in each of the three endothelial cell lines analyzed (Fig. 1A, top panel). Though equivalent numbers of cells were plated per well, the differences amongst the endothelial cell lines in titer at time zero were not surprising given that cell lines grow at varying rates and to varying densities. A comparison of viral titers at 36 hours revealed that the three endothelial cells lines produced titers comparable to those achieved in 3T3 fibroblast cells. Additionally, multi-step growth in MB114 and CD3 endothelial cells was similar to growth in 3T3 fibroblasts (Fig. 1A, bottom panel). These data demonstrated that γHV68 is capable of replication in endothelial cells infected *in vitro*.

Using a GFP-labeled virus, we next sought to determine if the percentage of infected endothelial cells was comparable to percentage of infected fibroblasts. Following infection of MB114 endothelial cells and 3T12 fibroblasts, cells were analyzed for expression of GFP from the γHV68 genome by flow cytometry. At 24 and 48 hours post-infection the majority of endothelial cells and fibroblasts expressed GFP (Fig. 1B). These data indicated that, like fibroblasts, endothelial cells were uniformly infected and supported the γHV68 lytic transcription program.

### A population of endothelial cells remained intact following γHV68 infection and uniformly expressed lytic antigens

**Figure 2:**
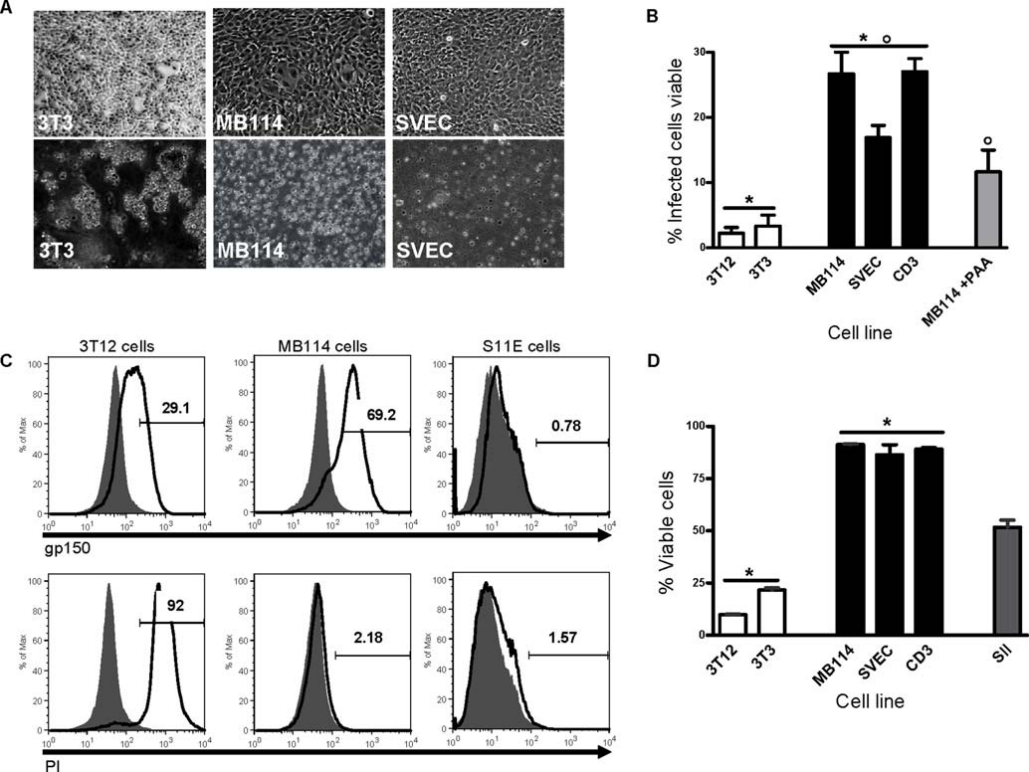
A population of endothelial cells remained intact following γHV68 infection. Non-adherent cells were collected at six days post-infection (MOI of 5 PFU/cell), cultured, and viability determined at six and 12 days post-infection. Status of γHV68 replication was determined at six days post-infection. (A) Bright field images of 3T3 fibroblasts, MB114 endothelial cells, and SVEC endothelial cells prior to infection (top panel) and at six days after γHV68 infection (bottom panel). Magnification of 200X. Infected cells were harvested by centrifugation and resuspended in fresh media prior to imaging. (B) Percentage of cells infected that were non-adherent and viable at six days post-infection. Where indicated, cells were treated with PAA after one hour of infection. Non-adherent cells were collected at six days post-infection and viability determined as percentage of trypan blue excluding cells. A significantly lower percentage of all fibroblast lines analyzed were viable at six days post-infection as compared to all endothelial cell lines analyzed (*p<0.001). In the presence of PAA, a significantly lower percentage of endothelial cells were non-adherent and viable at six days post-infection (°p = 0.018). Data is from 2–3 independent experiments per cell line. (C) Following collection at six days post-infection, cells were centrifuged and stained for surface expression of gp150 (top panel) or with propidium iodine (PI) (bottom panel). Latent S11E cells do not express lytic viral proteins and are a negative control for gp150 expression, as well as a positive control for viable cells. Fluorescence was determined relative to unstained cells (grey) and numbers reflect percent positive staining. Histograms are representative of two independent experiments. (D) Cultures of non-adherent cells at 12 days post-infection were stained with PI and viability determined by flow cytometry as percent PI negative cells. n = 3 per cell line. Cultures of endothelial cells remained significantly more viable than fibroblasts at 12 days post-infection (*p<0.001). S11s were analyzed as a positive control for viable cells.

Uninfected endothelial cells and fibroblasts grew as adherent monolayers (Fig. 2A, top panel). However, in infected endothelial cell cultures, we observed a population of intact, phase-bright, and non-adherent cells as early as 96 hours post-infection, whereas only cellular debris remained in the infected fibroblast cultures (data not shown). At six days post-infection, we collected non-adherent cells and cellular remains by centrifugation. After washing, we resuspended this material in complete media for culture and analysis. The cells harvested from the infected endothelial cultures appeared as individual, intact cells suspended in culture, whereas the material harvested from the infected fibroblast cultures appeared as clumps of cellular debris (Fig. 2A, bottom panel). Prior to centrifugation, we measured cell viability by trypan blue exclusion. Very few infected fibroblasts remained as viable, non-adherent cells at six days post-infection (Fig. 2B). However, a significant proportion of endothelial cells remained as viable, non-adherent cells at six days post-infection. When MB114 endothelial cells were treated with a non-toxic dose of phosphonoacetic acid (PAA), an inhibitor of viral DNA replication and late gene synthesis, most cells remained adherent, and there were significantly fewer detached, viable cells (Fig. 2B). PAA alone had no effect on the viability, adherence, or phenotype of uninfected MB114 cells in culture (data not shown). These data revealed that a population of endothelial cells became non-adherent and remained viable at six days post-infection, whereas fibroblasts were destroyed by γHV68 infection. Additionally, this outcome of infection in endothelial cells was influenced by late viral gene expression, as most cells remained adherent in the presence of PAA.

Next we determined the status of virus replication in the viable and non-adherent cells. Cells collected at six days post-infection were analyzed by flow cytometry for expression of the γHV68 glycoprotein, gp150, which is expressed during the lytic transcription program on the surface of infected cells [19]. Cell viability was also determined using propidium iodide (PI), a cell impermeant dye that is excluded from intact cell membranes. In these experiments we also included the S11E cell line, a mouse B cell lymphoma line which harbors latent γHV68, and therefore is negative for gp150 surface expression [20]. The majority of MB114 endothelial cells and 3T12 fibroblasts were positive for surface gp150 expression (Fig. 2C). While 3T12 fibroblasts were mostly PI positive, MB114 endothelial cells excluded PI. These data indicated that endothelial cells which remained viable at six days post-infection were undergoing the lytic transcription program and were not latent or uninfected.

**Figure 3:**
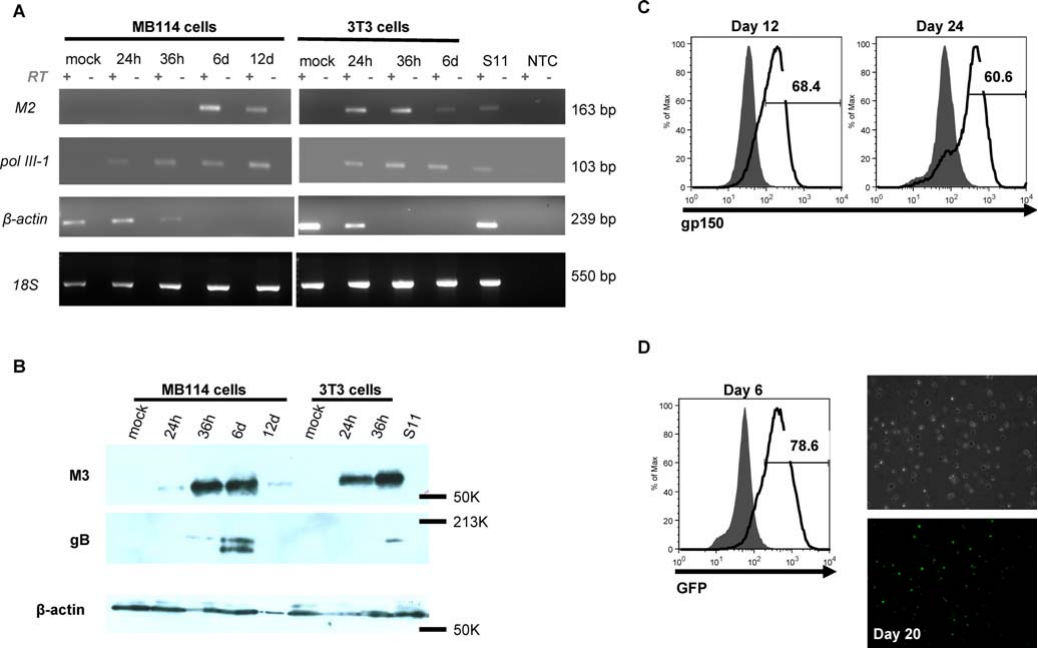
Endothelial cells which were viable after γHV68 infection were productively infected. (A) MB114 cells contained viral gene transcripts as far as 12 days post-infection. RT-PCR analysis of viral gene transcripts. 100 ng of total RNA from mock infected and infected MB114 and 3T3 cells and from S11 cells was added to each of the RT reactions along with primers specific for the viral transcripts polymerase III-1 and M2, and the cellular transcripts β-actin and 18S. No RT and no template controls are indicated. (B) MB114 cells contained viral proteins as far as 12 days post-infection. Immunoblot of lytic viral protein expression. 10 µg of total protein from mock infected and infected MB114 and 3T3 cells and from S11 cells were loaded per lane and blots probed with antibodies to the lytic γHV68 proteins M3 (top) and gB (middle), and mouse β-actin (bottom) as a loading control. Mock infected cells were collected at 24 hours. Latent S11 cells do not express lytic viral proteins and served as a negative control. (C) At the indicated times post-infection, MB114 cells were analyzed for gp150 surface expression by flow cytometry. Fluorescence was determined relative to unstained cells (grey). Histograms are representative of two independent infections. (D) Six days post-infection with GFP-γHV68, MB114 cells were collected and analyzed by flow cytometry for GFP expression. Cells were cultured and brightfield (top) and fluorescent (bottom) images taken at 20 days post-infection, 100X magnification. Fluorescence was determined relative to cells infected with wildtype γHV68 (grey), and histogram is representative of two independent infections.

At 12 days post-infection we determined the viability of cells collected and cultured at six days post-infection. PI staining of S11 cells, after six days in culture, revealed that 51.7% (±2.5 SEM) of cells were PI negative and thus viable. PI staining of post-infection fibroblasts, treated in parallel with infected endothelial cells, revealed that only 14.5% (±1.5 SEM) of the cells harvested remained intact. In contrast, PI staining of post-infection endothelial cells revealed that 92.5% (±0.3 SEM) of the cells harvested were intact and viable (Fig. 2D). Analysis of the viral replication program in MB114 endothelial cells at 12 days post-infection revealed that the majority of cells continued to express gp150 (Fig. 3C). Therefore, infection of endothelial cells with γHV68 resulted in a population of cells that escaped lysis and remained intact as far as 12 days post-infection, while undergoing the lytic transcription program.

### Intact endothelial cells were productively infected and underwent a continued lytic program

The observed viability of endothelial cells after γHV68 infection could be the result of escape from infection or latent infection of these cells. To determine the infection status of the intact endothelial cells, we performed RT-PCR analysis of viral gene transcripts. The γHV68 M2 gene transcript is synthesized during both lytic and latent infection, as are the polymerase III (pol III) transcripts encoded at the left end of the viral genome [21],[22]. Latently infected S11 cells contained both pol III-1 and M2 transcripts (Fig. 3A). We detected these transcripts in 3T3 fibroblasts at 24 and 36 hours post-infection, as well as from the 3T3 cellular debris harvested at six days post-infection, while no cellular β-actin transcript was detected from infected 3T3 cells at 36 hours post-infection. No RNA could be detected from infected 3T3 debris at 12 days post-infection, whereas we recovered RNA from infected MB114 cells as far as 12 days post-infection. Pol III-1 and M2 transcripts were detected in infected MB114 cells at 24 and 36 hours post-infection and from the intact, cultured cells at six and 12 days post-infection. We detected β-actin RNA in infected MB114 cells at 24 and 36 hours post-infection, but not at six and 12 days post-infection. However, we detected another cellular mRNA transcript, cyclophilin A, at six days post-infection in 3T3 fibroblasts and MB114 cells (Fig. S2), and comparable detection of the cellular 18S rRNA transcript was observed in all conditions. Gradual loss of the cellular β-actin transcript in viable cells was not surprising given that β-actin has been documented to vary significantly in the setting of virus infection [23], and selective degradation of certain mRNAs frequently occurs during virus infection [24]–[28]. Analysis of viral gene transcripts indicated that those endothelial cells which remained intact after γHV68 infection contained viral gene transcripts as far as 12 days post-infection.

To determine whether the intact, infected endothelial cells were undergoing active viral replication, we analyzed infected cells for the expression of early and late lytic replication-associated M3 and gB. As predicted, 3T3 fibroblasts, which support lytic replication, expressed both M3 and gB (Fig. 3B). In contrast, S11 B cells, which contain latent γHV68, did not express either M3 or gB. Intact, infected MB114 endothelial cells not only expressed M3 and gB early (36 hours), but also at six and 12 days post-infection. A faster migrating gB-specific band was also detected at six days post-infection, and may indicate differential glycosylation or degradation. Additionally, infected MB114 endothelial cells were positive for surface protein expression of gp150 at both 12 and 24 days post-infection (Fig. 3C). Our analysis of lytic protein expression indicated that endothelial cells which remained intact after γHV68 infection expressed early and late lytic proteins, indicating that not only did γHV68 infected endothelial cells still contain virus, these cells also expressed viral proteins indicative of active viral replication, and not latency.

To further test the status of viral replication in endothelial cells, we infected MB114 endothelial cells with GFP-γHV68 and collected the intact, non-adherent cells at six days post-infection. GFP expression is driven by the CMV immediate early promoter and has been demonstrated to be expressed only during lytic infection [29]. At the time of harvest, these cells uniformly expressed GFP (Fig. 3D histogram) and continued to do so at 20 days post-infection, (Fig. 3D micrographs). Therefore, the non-adherent endothelial cells which remained intact after γHV68 infection did not escape infection and were not latently infected.

### Intact, infected endothelial cells released new infectious virus particles

**Figure 4:**
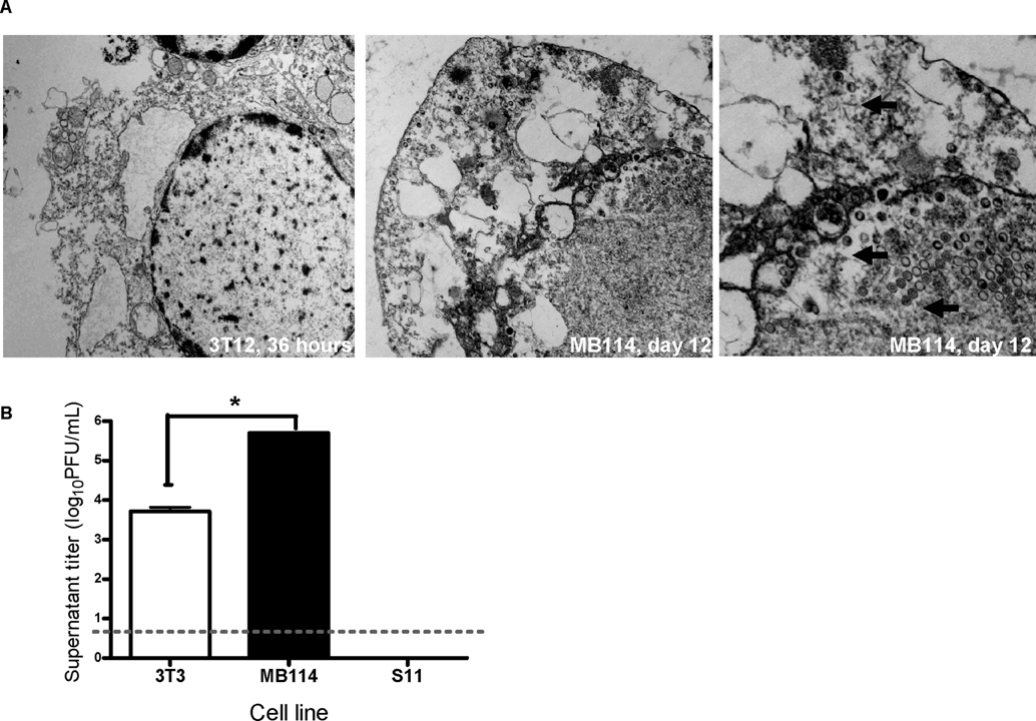
Viable γHV68-infected endothelial cells produced and released new infectious virus particles. (A) MB114 cells contained virions at various stages post-infection throughout the nucleus and cytoplasm. Transmission electron micrographs (TEM) of 3T12 cells and MB114 cells infected with MOI of 5. 36 hours post-infection, adherent 3T12 cells were collected and combined with non-adherent material in media (left panel). At six days post-infection, only non-adherent MB114 cells remained (middle and right panel). These cells were collected and cultured until 12 days post-infection. Pelleted cells were fixed in glutaraldehyde for TEM. Black arrows indicate virions. (B) MB114 cells released significantly more virus into the media as far as 12 days post-infection than 3T3 fibroblasts (p = 0.001). Non-adherent 3T3 fibroblasts and MB114 cells were collected at six days post-infection, washed twice, and resuspended in complete RPMI. At 12 days post-infection, cell-free supernatant titers were determined by plaque assay. Supernatant titer from latently infected S11 B cells, cultured in the same manner as infected 3T3 cells and MB114 cells was below the limit of detection for the plaque assay (0.5 log_10_ PFU/mL, indicated by dashed line).

After establishing that the intact endothelial cells harvested at six days post-infection were indeed infected, we determined whether these cells produced mature virions. Transmission electron microscopy (TEM) of these cells revealed virions at various stages of maturation throughout the nucleus and cytoplasm of 100% of the 20 cells imaged at 12 days post-infection (Fig. 4A). TEM analysis of 3T12 fibroblasts at 36 hours post-infection demonstrated that the cells were lysed by infection, with no intact membranes, and mature virions were not evident in any discernable subcellular structures. Unlike fibroblasts, endothelial cells which remained intact after γHV68 infection contained virions at various stages of maturation throughout the cytoplasm and nucleus at 12 days post-infection.

The culture conditions we established for maintaining the non-adherent cells included centrifugation and resuspension in fresh media every six days (Fig. S1). To test the contribution of non-adherent cells to virus production, aliquots of the supernatants were collected for measurement of cell-free virus. A culture of latently infected S11 cells did not yield any detectable cell-free virus after six days in culture by plaque assay (Fig. 4B). In contrast, after six days in culture (12 days post-infection), cell-free supernatant from infected MB114 cells yielded significant viral titer, and this titer was significantly higher than that of supernatant taken from infected 3T3 cultures. Therefore, endothelial cells continued to release new infectious virions for at least 12 days post-infection. Infected endothelial cell supernatant contained 100-fold more virus than that of a lysed, infected, fibroblast culture.

### Intact endothelial cells undergo transient proliferation in culture after γHV68 infection

**Figure 5:**
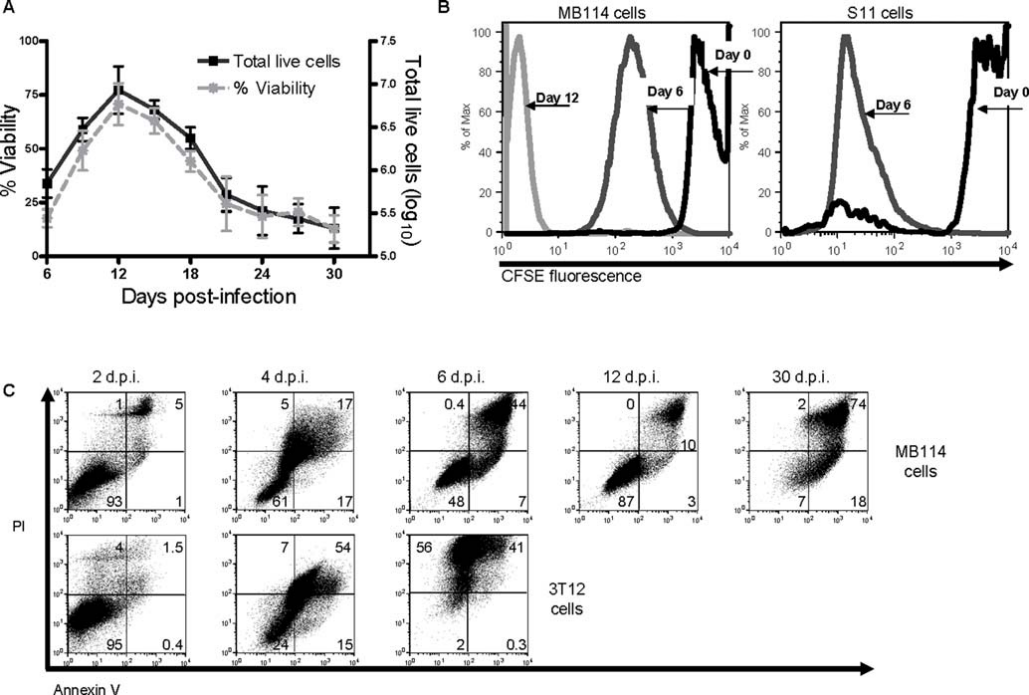
Endothelial cells exhibited prolonged viability and proliferated after γHV68 infection. Viability and proliferation of an endothelial cell line collected at six days post-infection (MOI of 5 PFU/cell) were determined every three days of culture. (A) Non-adherent MB114 endothelial cells collected at six days post-infection remained viable as far as 30 days post-infection. Viability (left axis) and live cell number (right axis) by trypan blue exclusion counts were measured every three days of culture. n = 3. (B) MB114 cells proliferated after infection. MB114 cells were stained with CFSE and analyzed by flow cytometry for green fluorescence prior to infection (day 0), and at six and 12 days post-infection. Unstained cells analyzed in parallel were consistent in autofluorescence throughout the analysis. S11 B cells, harboring latent γHV68 genome, were stained and analyzed in parallel as a positive control. n = 2 per cell line. (C) Analysis of viability in MB114 cells (top panel) and 3T12 cells (bottom panel) following γHV68 infection. Viability was determined by double staining of PI and annexin V, and gated on unstained cells. The results are representative of 2–4 independent experiments.

After the initial observation that γHV68 infection of several endothelial cell lines from different anatomic locations resulted in a population of cells that escaped lysis, we focused our analysis on this outcome in MB114 endothelial cells. Beginning at the time of collection (day six post-infection), we counted intact, trypan blue excluding cells every three days. Infected MB114 cells remained viable for at least 30 days post-infection (Fig. 5A).

During this course of infection, the number of intact cells in the culture increased from 7.08×10^5^ (±0.16 SEM) to 8.51×10^6^ (±0.28 SEM) during the first six days of culture, a ten-fold increase. A corresponding increase in the percentage of membrane viable cells also occurred during this time. Subsequent to day 12 post-infection, the total number of intact cells began to decrease, as did the overall viability of the culture (Fig. 5A). The observed increase in both intact cell number and percent viability during the first six days of culture, concurrent with positive gp150 staining (Fig. 2C and Fig. 3C), indicated that endothelial cells were intact and proliferating in culture while maintaining the lytic transcription program.

To further examine the proliferation observed during the first six days of culture, we stained MB114 cells with carboxyfluoroscein (CFSE) prior to infection. CFSE is a fluorescent molecule used to measure cell proliferation, in that each time a cell divides, the two daughter cells contain half the CFSE of the parent cell. S11 cells demonstrated a drop in CFSE signal intensity after six days in culture, consistent with cell division. At the time of harvest (day six post-infection), MB114 cells also demonstrated a drop in CFSE signal intensity consistent with multiple cell divisions (Fig. 5B). After six more days in culture (12 days post-infection) CFSE signal intensity dropped further, consistent with continued cellular proliferation. Thus, endothelial cells which remained intact after γHV68 underwent multiple rounds of proliferation.

Note that by 30 days post-infection, however, the viability of the culture was quite low in comparison to earlier time points (Fig. 5A). To further test the fate of the surviving endothelial cells, we examined viability of infected cells by staining with annexin V and PI (Fig. 5C). MB114 cells were 92.4% (±0.6 SEM) viable (annexin V negative, PI negative) at two days post-infection and 72.5% (±14.5 SEM) viable at four days post-infection. At six days post-infection all MB114 cells were non-adherent and 31.0% (±10.74 SEM) of these non-adherent cells were viable. In contrast to MB114 cells, dying cells dominated infected 3T12 cultures at six days post-infection These data indicate that many MB114 cells died early during infection, and in agreement with our proliferation data (Fig. 5A and 5B) the population of viable cells increased to 86.4% (±0.6 SEM) of the culture by 12 days post-infection. However, by 30 days post-infection dying cells finally dominated the infected culture. These data support that the subset of cells surviving and proliferating at six days post-infection were unique their extended survival, but did not remain viable indefinitely. Of significance, during the course of this infection most of the cells expressed the lytic glycoprotein gp150 on their surface at both early and late time points (Fig. 2C and Fig. 3C). Moreover, the percentage of cells expressing gp150 did not change markedly with time (Fig. 2C and Fig. 3C). These data indicate that the vast majority of viable, infected endothelial cells were undergoing active viral replication, and that these cells were neither latently infected nor had escaped infection.

### Intact endothelial cells were altered in size, shape, and surface marker expression after γHV68 infection

**Figure 6:**
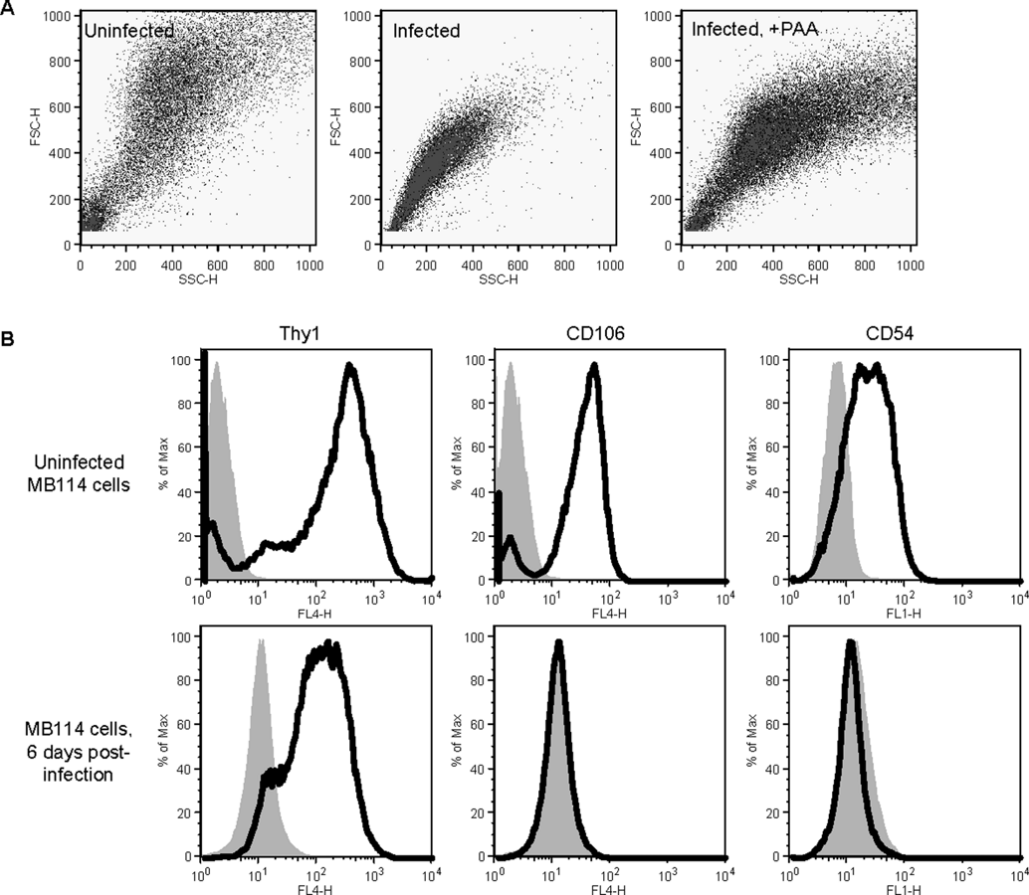
Infected and viable endothelial cells were altered in size, shape, and surface protein expression. Flow cytometric analysis of morphology and surface protein expression of non-adherent endothelial cells collected at six days post-infection. (A) MB114 cells were altered in size and granularity by γHV68 infection. Flow cytometric determination of forward and side scatter properties of MB114 cells collected at six days post-infection. MB114 cells treated with phophonoacetic acid (PAA) one hour after infection were collected and analyzed at six days post-infection. n = 2. (B) MB114 cells were altered in surface protein expression after γHV68 infection. Uninfected and infected MB114 cells harvested at six days post-infection were analyzed for cell surface expression of Thy1, ICAM-1, and VCAM-1 by flow cytometry. Fluorescence was determined relative to unstained cells (grey). Results are representative from three independent experiments.

To investigate the morphologic differences between uninfected endothelial cells and the endothelial cells harvested at six days post-infection, we quantified cell size and internal granularity by flow cytometric determination of forward versus side scatter. Uninfected MB114 cells exhibited a broad range of light scatter, indicative of a cell population diverse in size and internal granularity. In contrast, infected cells were very uniform in forward and side scatter (Fig. 6A). PAA treatment immediately following MB114 infection resulted in significantly fewer cells becoming non-adherent (Fig. 2B). Like the uninfected MB114 cells, MB114 cells treated with PAA following infection exhibited a broad range of forward and side scatter (Fig. 6A). Thus, γHV68 infection of endothelial cells resulted in a uniform population of cells with altered morphology, and this outcome was dependent on productive viral infection and late viral gene expression.

To further investigate the differences between uninfected endothelial cells and the intact cells harvested six days after infection, we compared the expression of cell-surface proteins on these two cell populations by flow cytometry. Because the cells harvested at six days post-infection persisted in culture as non-adherent cells, we chose to investigate surface expression of the adhesion markers intercellular adhesion molecule-1 (ICAM-1, CD54) and vascular cell adhesion molecule-1 (VCAM-1, CD106). Additionally, we analyzed cells for surface expression of Thy1, a cell surface protein which is upregulated on the surface of activated endothelial cells and functions in cell-cell interactions [30]–[32]. Uninfected MB114 cells expressed ICAM-1, VCAM-1, and Thy1 (Fig. 6B). In contrast, infected endothelial cells expressed neither ICAM-1 nor VCAM-1, but did express Thy1. Based on these data, γHV68 infected endothelial cells down-regulate cell surface expression of two adhesion molecules, while maintaining surface expression of an activation marker.

### In the absence of the viral cyclin, endothelial cell viability after γHV68 infection is reduced

**Figure 7:**
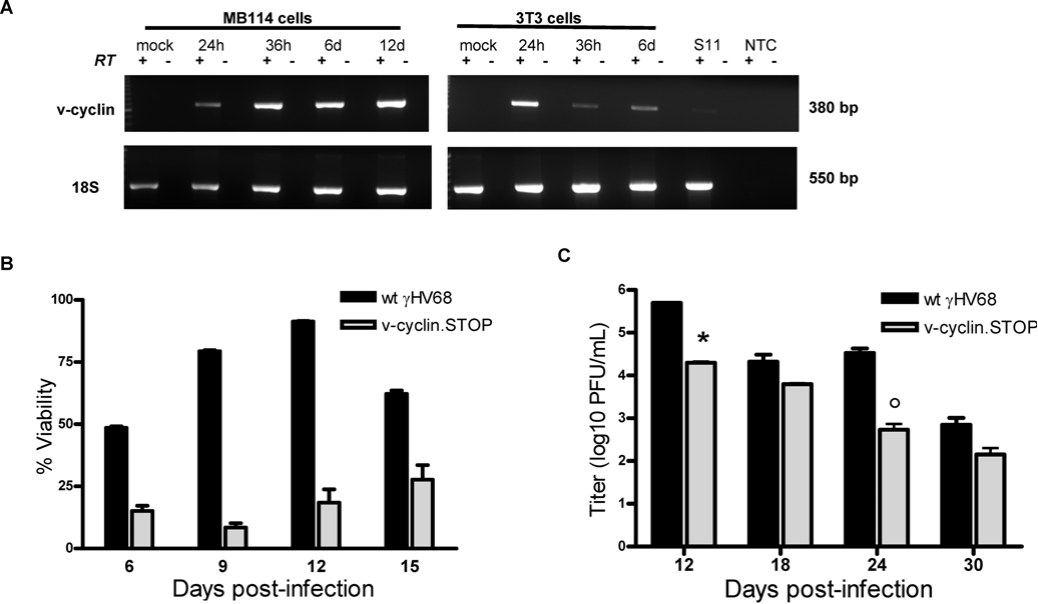
Optimal viability of endothelial cells after γHV68 infection requires the viral cyclin. (A) MB114 cells contained the γHV68 v-cyclin transcript. RT-PCR analysis of the γHV68 v-cyclin transcript. Total RNA was isolated from infected MB114 cells and 3T3 cells at the indicated times post-infection, as well as from latently infected S11 B cells. 100 ng of RNA from each sample was subjected to RT-PCR analysis with primers specific for the v-cyclin transcript. As a loading control, we also amplified the cellular transcript β-actin. No RNA could be isolated from cultured 3T3 cells at 12 days post-infection. No RT and no template controls are indicated. (B & C) Viability and persistent viral replication in the presence or absence of the v-cyclin. MB114 cells were infected with wildtype (black) or v-cyclin.STOP γHV68 (grey) at an MOI of 5 PFU/cell. Cells were harvested at six days post-infection by centrifugation from supernatant, and cultured in complete RPMI. Every six days cells were centrifuged to remove supernatant for titer (C) and stained with propidium iodine (PI) every three days (B). Viability of post-infection cultures was determined as percent PI negative cells by flow cytometry. Significant differences in viability were observed between wildtype γHV68 and v-cyclin.STOP infections at day six (p<0.001), day nine (p<0.001), day 12 (p<0.001), and day 15 (p = 0.002). n = 3 for wildtype infection and n = 4 for v-cylin.STOP infection. (C) Cell free virus titer of supernatants from wildtype γHV68 (black) and v-cyclin.STOP γHV68 (grey) infected cells was determined by plaque assay at the indicated times post-infection. Significant differences observed at day 12 (p = 0.008) and day 24 (p = 0.042). n = 2.

We demonstrated that γHV68 infection of endothelial cells resulted in a population of intact cells which persisted in culture as non-adherent cells and continued to produce new virus. Our analysis of endothelial cell infection in the presence of PAA indicated that this outcome is influenced by viral DNA replication and/or late gene synthesis (Fig. 2B and Fig. 4A). To begin dissecting the mechanism of persistent γHV68 in endothelial cells infected *in vitro*, we tested the role of the γHV68 viral cyclin (v-cyclin) in this system. We measured expression of the γHV68 v-cyclin in infected MB114 endothelial cells and infected fibroblasts by RT-PCR and western analysis. Notably, the γHV68 v-cyclin gene transcript and protein were detectable in infected endothelial cells and fibroblasts (Fig. 7A and Fig. S3A).

To determine the role of the v-cyclin in endothelial cell infection, we infected MB114 cells with either wildtype γHV68 or a v-cyclin deficient γHV68 (v-cyclin.STOP γHV68) [18]. Cells were harvested at six days post-infection and cultured as described previously. As per previously published reports in fibroblasts, we determined that the v-cyclin was dispensable for γHV68 growth in endothelial cell lines by multi-step growth assays (data not shown). Cultures of MB114 cells infected with wildtype virus were 48.5% (±0.61 SEM) viable at day six, 79.4% (±0.36 SEM) viable at day nine, 91.3% (±0.26 SEM) at day 12, and 62.2 % (±1.33 SEM) at day 15 post-infection. In contrast, cultures of MB114 cells infected with v-cyclin.STOP γHV68 were 15.1% (±2.093 SEM) viable at day 6, 8.4% (±1.767 SEM) viable at day 9, 18.4% (±5.304 SEM) viable at day 12, and 27.7% (±5.817 SEM) viable at day 15 post-infection. Therefore, in the absence of the v-cyclin, endothelial cell viability was significantly impaired after γHV68 infection.

Because viability of MB114 cells after infection was impaired in the absence of the v-cyclin, we next determined the effect of this viral gene on persistent viral replication in viable endothelial cells. Cell free supernatants from infected MB114 cells were titered by plaque assay (Fig. 7C). Infections with both wildtype and v-cyclin.STOP virus resulted in persistent viral production up to 30 days post-infection. Total viral titers from v-cyclin.STOP infections were significantly less than wildtype infections at 12 and 24 days, however, an equivalent amount of virus was produced per cell as in wildtype γHV68 infections. Cell surface protein expression of ICAM-1, VCAM-1, and Thy1 was the same following infection with either wildtype or v-cyclin.STOP virus (Fig. 6B and Fig. S3B), thus the v-cyclin was not required for the surface phenotype of infected endothelial cells and the surface phenotype of infected endothelial cells did not predict survival. Therefore, upon infection, the primary function of the v-cyclin was to promote the viability of endothelial cells.

### Primary endothelial cells were infected *in vivo*, and *ex vivo* demonstrated prolonged viability while supporting γHV68 growth

Given that immortalized endothelial cell lines are inherently different from endothelial cells *in vivo*, we next determined the outcome of γHV68 infection in primary endothelial cells. Primary endothelial cells were isolated from C57/BL6 mouse lungs and characterized as per previously published methods (Fig. S4).

**Figure 8:**
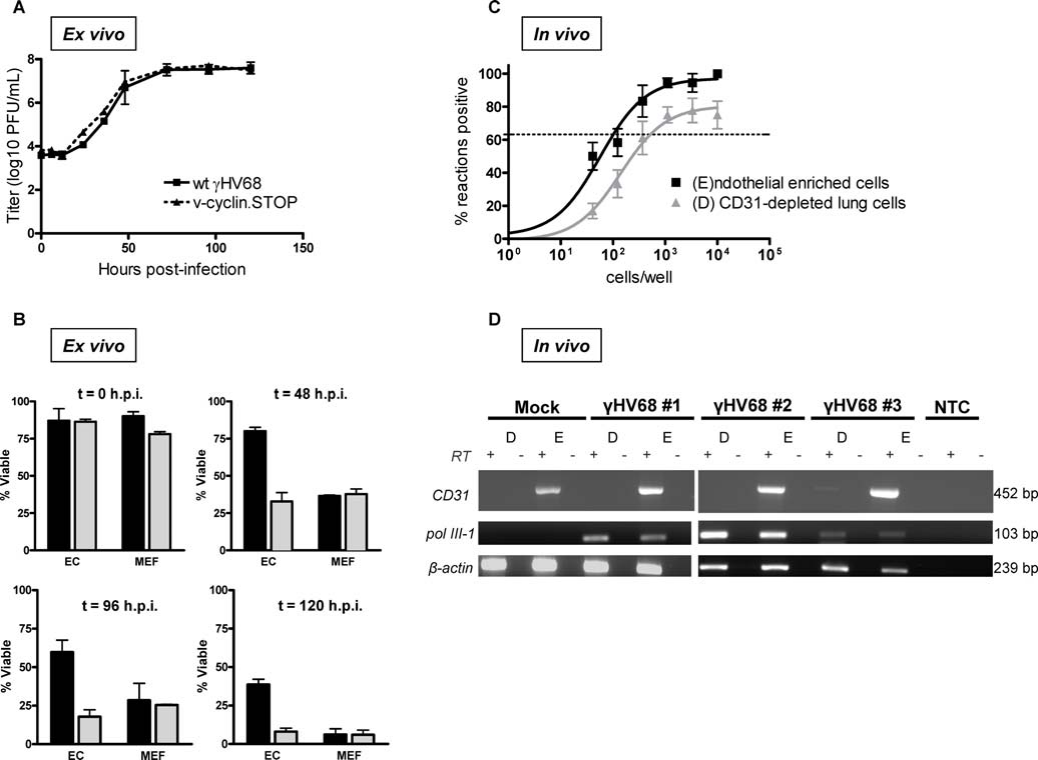
Primary endothelial cells were infected *in vivo*, and demonstrated prolonged viability while supporting γHV68 growth *ex vivo*. Multi-step growth assay of γHV68 in primary C57/BL6 lung endothelial cells (A) and corresponding cell viability (B). Primary cells were infected at an MOI of 0.05 PFU/cell with either wildtype γHV68 or v-cyclin.STOP γHV68, harvested at the indicated times, and trypan blue exclusion counts performed (B). (A) Samples were multiply freeze-thawed prior to quantification by plaque assay on NIH 3T12 cells. n = 3. (B) At 0, 48, 96, and 120 hours post-infection, trypan blue exclusion counts were performed on primary lung endothelial cells and primary MEFs infected with either wildtype or v-cyclin.STOP γHV68 at an MOI of 0.05 PFU/cell. n = 3. Limiting dilution-PCR of viral DNA (C) and RT-PCR (D) analysis of endothelial enriched (E) and depleted (D) lung cells from CD8-alpha knockout mice at six days post-intranasal infection. (C) Frequency of viral genome-positive cells was determined by LD-PCR. Percentage of positive PCR reactions are indicated on the y axis and the number of cells analyzed is indicated on the x axis. For each cell dilution, 12 PCR reactions were analyzed. The frequency of viral genome positive cells was determined by Poisson distribution indicated by the dashed line at 63.2%. Data represent the averages of three separate infected animals. Error bars represent standard errors of the mean. (D) Total RNA was isolated from D and E populations and 100 ng of RNA subjected to RT-PCR analysis with primers specific for the γHV68 pol III-1 transcript and the cellular transcripts CD31 and β-actin. Results are shown from three infected and one mock infected mouse.

First, we determined if primary lung endothelial cells could support growth of wildtype γHV68 by multi-step growth assay (Fig. 8A). Because of the apparent role for the v-cyclin in infection of endothelial cell lines, we also analyzed growth of v-cyclin.STOP virus in primary endothelial cells. Infection proceeded with kinetics comparable to previously published infection of NIH 3T12 fibroblasts [18]. Growth curves did not differ between wildtype and v-cyclin.STOP virus. These data revealed that primary endothelial cells supported growth of γHV68, irrespective of the v-cyclin.

Second, we analyzed the percent of cells that remained viable after γHV68 infection at a low MOI. Immediately following removal of the viral inoculum (t = 0), mouse embryonic fibroblasts (MEFs) and primary lung endothelial cells did not differ significantly in viability (p>0.05) (Fig. 8B). However, at 48 hours post-infection with wildtype γHV68, primary lung endothelial cells were significantly more viable than MEFs (p<0.001), and remained significantly more viable at 96 (p<0.05) and 120 hours post-infection (p<0.001). Notably, endothelial cells infected with the v-cyclin deficient γHV68 had reduced viability at 48, 96, and 120 hours post-infection. Given that cells were infected at a very low MOI, it was not surprising that a proportion of MEFs (28.5%±11.1 SEM) were viable at 96 hours post-infection. However, unlike endothelial cells, MEFs were mostly lysed by 120 hours post-infection with wildtype virus. These data demonstrate that primary endothelial cells surpass MEFs in viability following γHV68 infection, and that this outcome is promoted by the v-cyclin.

Lastly, we examined lung endothelial cells following acute γHV68 infection *in vivo*. Our *in vitro* studies revealed that viable, infected endothelial cells were not latently infected, but uniformly supported a lytic viral program (Fig. 2C, Fig. 3C and 3D). Because persistent viral infection and lytic viral antigen expression is likely to be cleared by an intact immune response, we analyzed endothelial cell infection in lung tissues of immune-deficient CD8-alpha null mice. At six days post-intranasal infection, lung cells enriched for those bearing the endothelial cell marker CD31 were analyzed alongside the remaining CD31 depleted lung cells. Given the possibility that a small percentage of non-endothelial cells can transiently express CD31 (i.e. macrophages and neutrophils), we depleted the CD31 positive cell population of these potential contaminating cells and analyzed resultant cell populations by flow cytometry (Fig. S5B). PCR analysis with single copy sensitivity for γHV68 gene50 (Rta) was performed to determine the frequency of viral genome positive cells in the CD31-enriched and remaining CD31-depleted lung cell populations (Fig. 8C). Data from this analysis revealed that approximately 1/102 CD31-enriched cells were viral genome positive, whereas 1/525 remaining CD31-depleted lung cells contained viral DNA. Thus, these data support that a surprisingly large proportion of lung endothelial cells were viral genome positive following *in vivo* infection.

To further demonstrate that these viral genome positive cells are actively infected, and to exclude the possibility of endocytic uptake of virus or abortive infection, we performed RT-PCR analysis on endothelial enriched and depleted lung cells (Fig. 8D). The transcript for the endothelial cell marker CD31 was robustly detected in the endothelial enriched lung cells from each of the four mice analyzed, whereas a very low level of this transcript was detected from the CD31 depleted lung cells of only one out of the four mice analyzed. In combination with flow cytometric analysis (Fig. S5B), these data support that our enrichment strategy was effective in isolating CD31+ cells from total lung cells. Additionally, the viral pol III-1 transcript was detected from both endothelial cell enriched and depleted lung cells of γHV68 infected mice, and was absent from lungs of mock infected mice. Detection of viral gene transcripts from lung cells indicates virus infection of these cells. Notably, within each infected mouse, pol III-1 detection was comparable between endothelial enriched and CD31-depleted lung cell populations, suggesting that detection of this viral transcript in the endothelial cell population was not due a few contaminating infected non-endothelial cells. Taken together, these data demonstrate γHV68 infection of endothelial cells *in vivo*.

## Discussion

Herpesviruses have been implicated as potential initiators of a variety of endothelial pathologies [1]–[4]. Given the intimate interactions observed between herpesviruses and endothelial cells, and the systemic spread of γHV68 during infection, we characterized the outcome of endothelial cell infection with γHV68, a small animal model for the human gammaherpesviruses. While γHV68 replicated comparably in endothelial cells and fibroblasts up to 36 hours post-infection, infected cultures of endothelial cells had a high percentage of viable, non-adherent cells that remained following infection. Of significance, these viable, non-adherent cells had not simply escaped infection, but instead were actively infected, as determined by the presence of multiple markers of the lytic replication program. While the absolute number of viable, infected endothelial cells varied among different cell lines and primary cells, the prolonged viability of endothelial cells in comparison to fibroblasts was remarkably consistent in endothelial cell lines from distinct anatomic locations, as well as in primary endothelial cells. Furthermore, optimal survival of both endothelial cell lines and primary endothelial cells was dependent on the presence of the γHV68 v-cyclin, indicating that this outcome was a process actively promoted by virus infection.

This conserved outcome of endothelial cell infection is particularly striking given that endothelial cells display phenotypic heterogeneity in structure and function depending on anatomic location [33]–[35]. Based on the persistent infection observed in diverse endothelial cell lines, and heightened viability of primary endothelial cells following infection, we propose that γHV68 may have evolved machinery to specifically promote persistent infection in endothelial cells.

Endothelial cells serve as a natural site of infection and possible viral reservoir of HCMV [36]–[40], suggesting a role for HCMV-infected endothelial cells in viral spread and persistence. Additionally, recent reports implicate circulating endothelial progenitor cells as potential reservoirs of KSHV and possible precursors of KS spindle cells [41],[42]. However, the specific mechanisms by which infected endothelial cells contribute to the pathogenesis of these human viruses remains unclear. Murine γHV68 pathogenesis involves dissemination from the lung to lymph nodes, spleen, and peritoneum [12],[15]. In light of this systemic spread, γHV68 likely encounters an endothelial cell barrier.

The human gammaherpesvirus KSHV causes a serious endothelial cell malignancy, KS, which predominantly occurs in immune-compromised individuals (e.g. AIDS patients). KS tumors are comprised of distinctive spindle cells of endothelial origin and a variable inflammatory infiltrate [43]–[46]. KSHV is detected primarily in the endothelial component of the lesion, and though most of these cells harbor latent KSHV, a subset of them enter the lytic cycle [47],[48]. While there is precedent for a mixed infection type within KS tumors (i.e. both lytic and latent infection), the history of the lytically infected cells remains elusive, though recent reports point to circulating endothelial progenitor cells [41],[42]. *In vitro*, endothelial cell infection with KSHV is predominantly latent [43], [49]–[51]. However, when these cells are transferred into mice, they show evidence of lytic gene expression and virus production [52].

Because viral DNA replication and/or late gene synthesis were important for endothelial cell outcome of γHV68 infection (Fig. 2B and Fig. 6A), our findings indicate that an active viral process occurred within the cells to yield these changes. Consistent with this hypothesis, we identified that the γHV68 v-cyclin is required for optimal endothelial cell viability after γHV68 infection *in vitro*. The γHV68 v-cyclin promotes cell cycle progression in primary lymphocytes and can function as an oncogene in transgenic mice [18]. While the v-cyclin is critical for reactivation from latency, to date the v-cyclin has been dispensable in all assays of lytic replication *in vitro* [53],[54]. Given the lytic nature of endothelial cell infection, the contribution of the v-cyclin to optimal endothelial cell viability after γHV68 infection indicates a role for the v-cyclin outside of its requirement in reactivation from latency. Additionally, the apparent role of the v-cyclin in this prolonged infection of both endothelial cell lines and primary lung endothelial cells may explain the slight decrease in lung titers that resulted in mice infected with low doses of v-cyclin.STOP γHV68 and other v-cyclin mutant viruses compared to wildtype γHV68 [55]. Although the precise mechanism by which the v-cyclin promotes endothelial cell viability is unknown at this time, it is possible that the v-cyclin provides growth cues that allow for anchorage-independent growth of endothelial cells after γHV68 infection.

While our initial analysis focused on the role of the v-cyclin, it is very likely that additional viral genes facilitate persistent endothelial cell infection. Lead candidates include the anti-apoptotic viral bcl-2 gene M11 and the viral GPCR (ORF 74), whose homologs in KSHV have been implicated in endothelial cell survival and transformation [56]–[59]. Additional candidates for optimal endothelial cell infection are the ribonucleotide reductase homologs, ORF 60 and 61 of γHV68. Although the role of these genes in γHV68 infection of endothelial cells is currently untested, the MCMV ribonucleotide reductase homolog is required for *in vivo* replication and pathogenesis of this endothelial cell-tropic virus [60].

One of the most noticeable alterations of the persistently infected endothelial cells described here is the extent of change in their cellular morphology and properties. Though we did not test the oncogenic potential of these cells, endothelial cells achieved anchorage-independent growth, a property frequently associated with oncogenic transformation. These cells also underwent significant changes in protein expression on the cell surface, with down-regulation of the cellular adhesion proteins ICAM-1 (CD54) and VCAM-1 (CD106). While there was decreased expression of ICAM-1 and VCAM-1, changes in cell surface expression were not global, since Thy1 expression remained positive on these cells (Fig. 6B). It is worth noting that infection of endothelial cells with KSHV results in down-regulation of MHC class I, PE-CAM (CD31), and ICAM-1 (CD54), but not LFA-3 (CD58) or Fas (CD95), and the viral genes K3 and K5 have been demonstrated to regulate this outcome [61]–[66]. The contribution of the mK3 gene of γHV68 to persistent endothelial cell infection remains untested. Down-regulation of certain surface markers but not others during viral infection indicates a specific phenomenon, rather than global down-regulation, and provides further evidence that an active process was responsible for the observed endothelial cell outcome of γHV68 infection.

While γHV68 may have evolved mechanisms that promote persistent infection of endothelial cells (e.g. the v-cyclin), it is also possible that endothelial cells have a cellular program that limits the cellular lysis typically observed during productive infection. This putative cellular adaptation may be particularly important in limiting destruction of blood vessel integrity during viral infection. A recent report of host cell response to γHV68 in three different cell types (fibroblasts, endothelial precursor cells and macrophages) identified 148 genes whose expression was altered in endothelial precursor cells, but not macrophages or fibroblasts [67]. Taken together with the unique endothelial cell outcome of γHV68 infection reported here, these data make a compelling argument for cell type specific responses to productive γHV68 infection.

We have provided evidence for γHV68 infection of endothelial cells *in vivo* at early times post-infection. Interestingly, infected cells (including endothelial cells) were not abundant in acutely infected lung tissue, as little to no GFP was detected by flow cytometric analysis of unfractionated lung cells after infection with a GFP-marked virus (data not shown), but required sensitive PCR methods for detection. Our *in vitro* analysis demonstrated a change in surface phenotype of infected endothelial cells which could correspond to substrate detachment and release into circulation. This is an intriguing idea in light of published reports of circulating endothelial cells and progenitor endothelial cells in virus infection. Our *in vitro* studies implicated endothelial cells as a persistent source of virus production, however, the extent to which endothelial cells contribute to γHV68 persistence *in vivo*, and to what degree it might be influenced by host immune status, are important issues that remains to be addressed. While it is unlikely that an intact immune response would permit such long-term expression of viral antigens *in vivo*, we hypothesize that persistently infected endothelial cells could provide a significant source of continued virus replication in immune-compromised individuals (i.e. AIDS patients), a context in which gammaherpesvirus-associated pathology frequently occurs.

Given the unusual properties of these viable and infected endothelial cells (e.g. anchorage-independent growth and altered cell surface protein expression), it will be important to critically address the potential role of endothelial cells as a reservoir for infection *in vivo* in both immune-competent and immune-deficient individuals.

In conclusion, our data provide evidence for prolonged gammaherpesvirus infection in endothelial cells. This outcome appears to be the result of a specific interaction between γHV68 and endothelial cells, as it is promoted by a viral gene (the γHV68 v-cyclin) and, to date, is unique to endothelial cells. These data further refine the concept of gammaherpesvirus infection and demonstrate that a gammaherpesvirus can undergo robust productive replication in the context of prolonged host cell viability. Identification of intermediate outcomes of gammaherpesvirus infection, such as the one we have described here, has major implications for our understanding of the nature of gammaherpesvirus infection as it relates to specific cell types. Such a course of infection provides an additional mechanism, beyond latency, by which gammaherpesviruses can achieve long-term propagation.

## Materials and Methods

### Viruses and tissue culture

Mouse fibroblast cell lines 3T3 (ATCC CRL-1658) and 3T12 (ATCC CCL-164) and mouse endothelial cell lines MB114 [68], SVEC4-10 (ATCC CRL-2181), and CD3 [69] were cultured in Dulbecco's Modified Eagle Media (DMEM) supplemented with 5% FBS (Hyclone, Logan, UT), 2 mM L-glutamine, 10 U/mL penicillin, and 10 µg/mL streptomycin sulfate. S11 [20] and S11E tumor cells [22] were cultured in RPMI 1640 medium (Gibco) supplemented with 10% FBS, 50 µM β-mercaptoethanol, 1 mM sodium pyruvate, 2 mM L-glutamine, 10 U/mL penicillin, and 10 µg/mL streptomycin sulfate (complete RPMI). Mouse embryonic fibroblasts were isolated from C57/BL6 mice as previously described [70] and cultured in DMEM supplemented with 10% FBS, 2 mM L-glutamine, 10 U/mL penicillin, 10 µg/mL streptomycin sulfay7te, and 250 ng/mL fungizone. Isolation, characterization, and culture of primary endothelial cells from C57/BL6 mice was done according to previously published methods [71] and is described in Protocol S1, Text S1 and Figure S3A.

γHV68 WUMS (ATCC VR-1465) and all recombinant viruses were grown and titered as previously described [72]. ΔK3TET^−^ γHV68 (γHV68-GFP) containing a GFP cassette under the control of an immediate early CMV promoter was generated and characterized by Dr. Phillip Stevenson [73]. γHV68 containing a stop codon within ORF 72 (v-cyclin.STOP. γHV68) was previously described [18].

All infections were carried out at a multiplicity of infection (MOI) of 5 plaque forming units (PFU) per cell. Inoculum was removed after one hour of infection at 37°C, cell monolayers rinsed three times with sterile phosphate buffered saline (PBS), and complete media added. Intact and non-adherent cells were collected at six days post-infection, at which time cells and media were collected, counted to determine post-infection viability by trypan blue exclusion, and then centrifuged for 10 minutes at 208×g. Cell pellets were washed twice in sterile PBS and then resuspended in complete RPMI at a concentration of 5×10^5^ viable cells/mL for continued culture. Cells were counted every three days of culture, and adjusted to a concentration of 5×10^5^ viable cells/mL. Every six days of culture, cells were counted and centrifuged for 10 minutes at 208×g to remove cell-free γHV68 and cellular debris. Pellets were washed twice in sterile PBS and resuspended in RPMI at a concentration of 5×10^5^ viable cells/mL. Culture scheme is depicted in supplement (Fig. S1).

### Viral titer by plaque assay

To measure virus replication, infected samples were analyzed by plaque assay at various times post-infection. To measure cell-free virus titer from infected cultures, supernatants were collected every six days of culture and analyzed by plaque assay. Samples were thawed, serially diluted, and plated onto NIH 3T12 cells in 12 well plates in triplicate. Infection was performed at 37°C for one hour. Cells were overlaid with a 1∶1 mix of DMEM and carboxymethylcellulose plus fungizone (final concentration 250 ng/mL). Plates were incubated for seven days at 37°C. On day seven, carboxymethylcellulose was removed, and plates were stained with 0.35% methylene blue in 70% methanol and rotated for 15–20 minutes, before rinsing with water and counting on a light box. All titers were determined in parallel with a laboratory standard.

### Flow cytometry

For propidium iodine (PI) viability studies, cells were washed twice in PBS (five minutes, 1000×g), resuspended in a 0.5 µg/mL PI solution, and incubated for 15 minutes. Following incubation, cells were centrifuged (five minutes, 1000×g) and washed in a solution of PBS, 2% fetal calf serum, and 0.1% NaN_3_ (buffer A). Cells were fixed in 1% paraformaldehyde, and analyzed by flow cytometry. For two parameter viability analysis, cells were washed in 1X annexin V binding buffer (BD Bioscience, San Jose, CA), resuspended in 0.5 µg/mL PI and 5 µL annexin V-FITC (BD Bioscience), incubated for 15 minutes, washed in binding buffer, fixed in 1% paraformaldehyde, and analyzed by flow cytometry.

For carboxyfluorescein (CFSE) proliferation studies, MB114 cells and Sll cells were washed twice in PBS (five minutes, 1000×g) and resuspended in a solution of PBS and 2% fetal calf serum (buffer B) at a concentration of 1×10^6^ cells per mL. An equal volume of 4 µM CFSE in buffer B was added to the cell suspension (2 µM final concentration) and pipet mixed. After three minutes the labeling reaction was quenched with an equal volume of fetal calf serum for 30 seconds, and buffer B was added for a total volume of 50 mL. Cells were centrifuged (five minutes, 1000×g), resuspended in buffer B, and aliquots collected for day 0 analysis. Remaining, labeled cells were resuspended in complete media and cultured. Labeled MB114 cells were infected at an MOI = 5 PFU/cell. Infected cells were harvested as described at day six post-infection and analyzed by flow cytometry at six and 12 days post-infection. Stained and unstained S11 cells were analyzed at the same time as a positive control.

For surface marker staining, cells were washed twice in buffer A (five minutes, 1000×g) and resuspended in primary antibody (1∶200 in buffer A, 25% 24G2 [74]). The following primary antibodies were used: CD106-biotin (Rat, IgG2a κ, clone 429 (MVCAM.A)), CD54-FITC (Armenian hamster, IgG1 κ, clone 3E2), Thy1.2-APC (Rat, IgG2a κ, clone 53-2.1) (BD Bioscience), monoclonal mouse anti-γHV68 gp150 (mouse IgG2a, a kind gift from Dr. Phillip Stevenson) [75]. Cells were then incubated for 45 minutes at room temperature. Following incubation, cells were centrifuged twice in buffer A (five minutes, 1000×g), resuspended in either buffer A or secondary staining reagent in buffer A (1∶500), and incubated for 20–30 minutes at room temperature. Secondary staining reagents were either streptavidin-APC (BD Bioscience) or anti-mouse IgG2a-FITC (Rat IgG1 κ, clone R19-15) (BD Bioscience). Cells were centrifuged twice in buffer A (five minutes, 1000×g) and analyzed by flow cytometry.

For forward versus side scatter analysis, MB114 cells harvested at six days post-infection were analyzed. Uninfected MB114 cells were detached from flasks with 0.5 mM EDTA and analyzed in parallel with infected cells. In certain experiments, to determine the effect of viral DNA replication and/or late gene synthesis on forward and side scatter properties, MB114 cells were treated with 200 µg/mL phosphonoacetic acid (PAA) after one hour of infection. At six days post-infection, non-adherent cells were harvested and counted by trypan blue exclusion to determine the percent of cells infected that were viable and non-adherent at time of harvest. Cells which remained adherent to the flask were then detached with 0.5 mM EDTA, combined with the harvested non-adherent cells, and analyzed by flow cytometry for forward and side scatter properties. Effective block of late gene synthesis was confirmed by plaque assay titer of treated cells. Viral titer was reduced 96.2% after 24 hours and 99.9% after six days as compared to untreated cells. 10 micron control beads were run in parallel with each experiment (Beckman Coulter, Fullerton, CA).

### Western analysis

Cells were collected and boiled in Laemmli buffer for 10 minutes. Total protein concentration of each cell extract was determined by Lowry assay (DC protein assay kit, Bio-Rad, Hercules, CA). 10–20 µg of total protein per extract was separated by sodium dodecyl sulfate-polyacrylamide gel electrophoresis. Proteins were electrotransferred (ThermoFisher Scientific, Portsmouth, NH) to PVDF membranes (Millipore, Billerica, MA) and blocked in PBS with 0.05% Tween-20 and 5% nonfat milk for one hour at room temperature. Western blots for γHV68 protein expression were incubated with 10 µg/mL monoclonal mouse anti-γHV68 gB [76] (kind gift of Dr. Phillip Stevenson), monoclonal mouse anti-γHV68 M3 at 1∶50, polyclonal rabbit anti-γHV68 v-cyclin at 1∶2000 (kind gifts of Dr. Herbert Virgin), and monoclonal antibody to mouse β-actin at 1∶1000 (Sigma Chemical, St. Louis, MO). Blots were washed for 45 minutes in PBS with 0.05% Tween-20, then incubated with donkey anti-mouse or donkey anti-rabbit antibodies at 1∶6000 for one hour. Blots were then washed in PBS containing 0.1% Tween-20 for 45 minutes. Proteins were visualized using an ECL Plus Western blotting detection kit (Amersham Pharmacia Biotech).

### RNA Isolation and RT-PCR Amplification

Total RNA was extracted from S11 cells, infected NIH 3T3 cells, infected MB114 cells, and murine lung cell fractions using *mir*VANA™ miRNA Isolation kit (Ambion, Austin, TX), per manufacturer's instructions. Amplifications were conducted using the following primer sets: γHV68 polIII-1 forward 5′ CAA CAG GTC ACC GAT CC 3′, γHV68 polIII-1 reverse 5′ GGA AGT ACG GCC ATT TC 3′, γHV68 M2 forward 5′ TAA GGA CCT CGT AGA GAT TGG C 3′, γHV68 M2 reverse 5′ ACG TTA AAG TCC CCA TGG AAG C 3′, γHV68 v-cyclin forward 5′ ATT AGC ACT GGG CGT TTC ATG 3′, γHV68 v-cyclin reverse 5′ GAC CTC CGT CAG GAT AAC AAC 3′, mouse β-actin forward 5′ GCC ACC AGT TCG CCA TGG 3′, mouse β-actin reverse 5′ CAA TGC CAT GTT CAA TGG GGT A 3′, mouse 18S forward 5′ AGA TCA AAA CCA ACC CGG TGA 3′, mouse 18S reverse 5′ GGT AAG AGC ATC GAG GGG GC 3′, mouse cyclophilin A forward 5′ ATT TGG CTA TAA GGG TTC CTC 3′, mouse cyclophilin A reverse 5′ ACG CTC CAT GGC TTC CAC AAT 3′, mouse CD31 forward 5′ AGG GGA CCA GCT GCA CAT TAG G 3′ and mouse CD31 reverse 5′ AGG CCG CTT CTC TTG ACC ACT T 3′. RT-PCR reactions were carried out using the OneStep RT-PCR Kit (Qiagen, Valencia, CA) containing a final concentration of primer at 0.5 µM. 100 ng of RNA was treated with RQ1 RNase-Free DNase (Promega, Madison, WI) prior to addition of the RT-PCR reaction mix. RT-PCR reactions were performed as follows: 30 min at 50°C (reverse transcription); 15 min at 95°C (heat inactivation of reverse transcriptase and activation of Taq polymerase); 35–40 amplification cycles of 30 sec at 95°C followed by 30 seconds of 48°C–50°C (dependent on the Tm of the primer set) followed by one min at 72°C and a single incubation for 10 minutes at 72°C. No template and no RT controls were included for each PCR and were negative.

### Transmission electron microscopy

Cells were pelleted (five minutes, 208×g), resuspended in 1 mL PBS, and pelleted again (five minutes, 208×g). Supernatant was removed and 2.5% glutaraldahyde solution (adjusted to pH 7.4 using HCl and to 400 mOsm using CaCl_2_) added. Samples were processed and imaged by Dr. Gary Mierau, The Children's Hospital Department of Pathology/Laboratory Services, Aurora, CO. Briefly, samples were post-fixed in 2% cacodylate buffered osmium tetroxide (pH 7.4), dehydrated in a graded series of alcohols, and embedded in epoxy resin. Sections, approximately 80 nm in thickness, were stained with uranyl acetate and lead citrate prior to examination at 60 kV with a Zeiss EM-10 transmission electron microscope (Carl Zeiss Inc, Thornwood, NY).

### Enrichment of lung endothelial cells from infected mice

Lung tissues were removed from CD8-alpha knock-out mice six days post-intranasal inoculation of 1×10^6^ PFU γHV68. Tissues were enzymatically digested and endothelial cells purified using the endothelial cell marker CD31 (PECAM-1) (Fig. S4A). Briefly, cells were stained with the following antibodies: anti-CD31-biotin (PECAM-1, clone MEC 13.3, rat IgG2a κ) (BD Bioscience), anti-CD45-PE (B220, clone RA3-6B2, Rat IgG2a κ) (BD Bioscience), anti-F4/80-PE (clone BM8, Rat IgG2a κ) (eBioscience, San Diego, CA), anti-CD4-PE (L3T4, clone RM4-5, Rat IgG2a κ) (eBioscience), anti-CD8a-PE (Ly-2, clone 53-6.7, Rat IgG2a κ) (BD Bioscience), and anti-Ly-6G and Ly-6c-PE (Gr-1, clone RB6-8C5, Rat IgG2b κ) (BD Bioscience). Cells were magnetically labeled with Anti-Biotin MultiSort beads, separated with OctoMACS separation unit, and released from the MultiSort beads as per manufacturer's instructions (Miltenyi Biotec Inc., Auburn, CA). Released cells were incubated with Anti-PE MultiSort beads and depleted of contaminating PE positive cells by magnetic separation. Flow cytometric analysis was performed to confirm flow through fraction from PE column as enriched in CD31 positive cells and depleted of PE positive cells (Fig. S5B).

### Limiting dilution nested PCR detection of γHV68 genome-positive cells

The frequency of lung cells from CD8-alpha knock-out mice containing γHV68 genome was determined using a previously described nested PCR assay (LD-PCR) with single-copy sensitivity to detect gene 50 of γHV68 [77]. Briefly, freshly isolated cells were counted, resupsended in isotonic solution, and then diluted in 10^4^ uninfected NIH 3T12 cells prior to serial dilution plating. Plated cells were lysed overnight in proteinase K, followed by two rounds of nested PCR. Reactions were performed on 12 replicates per dilution per sample and products resolved on a 2% agarose gel and identified by ethidium bromide staining. PCR sensitivity was quantitated using 10, 1, or 0.5 copies of a gene 50 containing plasmid (p*Bam*H I N) diluted in 10^4^ uninfected NIH 3T12 cells.

### Statistical analysis

Data were analyzed using GraphPad Prism software (GraphPad Software, San Diego, CA). Data were analyzed using the paired Student's *t* test to determine statistical significance. Limiting dilution data were subjected to nonlinear regression analysis, and the frequency of genome-positive cells was calculated using the Poisson distribution to assume that the cell number at which 63.2% of events were detected corresponded to the occurrence of a single event.

### Accession numbers

PECAM-1: NP_001027550; NM_001032378

ICAM-1: NP_034623; NM_010493

VCAM-1: NP_03523; NM_011693

β-actin: NP_031419; NM_007393

Thy1: NP_033408; NM_009382

Rn18s: NR_003278

CypA: NP_032933; NM_008907

γHV68 WUMS complete genome: U97553

γHV68 v-cyclin: AAB66456

γHV68 gB: AAB06229; U08990

γHV68 gp150: AAC42214; L47321

γHV68 M2: AAF19270

γHV68 M3: AAF1271; AF127083

γHV68 ORF50: NC_001826.2; NP_04487.1

## Supporting Information

Text S1 (0.03 MB DOC)

Protocol S1 Isolation and characterization of endothelial cells from murine lung tissue.
(0.03 MB DOC)

Figure S1 Schematic of conditions for culturing non-adherent cells following γHV68 infection. Non-adherent cells collected at six days post-infection, MOI = 5PFU/cell were cultured as described in Materials and Methods.
(0.04 MB TIF)

Figure S2 RT-PCR analysis of cyclophilin A transcript in infected cells. 100 ng of total RNA from mock infected and infected MB114 and 3T3 cells was added to each RT reaction along with primers specific for the cellular transcript cyclophilin A. No RT and no template controls are indicated.
(1.88 MB TIF)

Figure S3 Cellular and viral protein expression following γHV68 infection. (A) MB114 cells contained viral proteins as far as 12 days post-infection. Immunoblot of γHV68 v-cyclin protein expression. 20 µg of total protein from mock infected and infected MB114 and 3T3 cells and from S11 cells were loaded per lane and blots probed with antibodies to γHV68 v-cyclin (top) and mouse β-actin (bottom). Mock infected cells were collected at 24 hours. Latent S11 cells do not express lytic viral proteins and served as a negative control. (B) The viral cyclin is not required for surface protein expression changes on infected endothelial cells. MB114 cells harvested at six days post-infection with v-cyclin.STOP γHV68 were analyzed for cell surface expression of Thy1, ICAM-1, and VCAM-1 by flow cytometry. Fluorescence was determined relative to unstained cells (grey). Results are representative from two independent experiments.
(2.98 MB TIF)

Figure S4 Isolation and characterization of primary murine lung endothelial cells for *ex vivo* culture. (A) Primary murine lung endothelial cells were isolated for *ex vivo* studies as described in Protocol S1. (B) Light micrographs of primary lung endothelial cells following isolation (top panel, 40X) and at confluence (bottom panel, 10X). (C) Analysis of cell surface protein expression in 3T3 fibroblast cell lines (negative control, left panel), CD3 lung endothelial cell lines (positive control, middle), and primary lung cells (right panel) by flow cytometry. Endothelial cell specific markers included CD31 and CD54. CD80 and CD86 were included as non-endothelial cell specific markers, though a previous report has identified low level CD80 expression on primary murine lung endothelial cells [71]. Fluorescence was determined relative to unstained cells (grey). Cell morphology and surface expression was similar to previously characterized primary endothelial cells [71].
(0.89 MB TIF)

Figure S5 Isolation and characterization of murine lung endothelial cells infected *in vivo*. (A) Primary murine lung endothelial cells were isolated for analysis of infection *in vivo* as described in Materials and Methods. Lung cells were stained for the endothelial cell marker CD31, as well as with a cocktail of PE labeleled antibodies specific to the following cell types (which includes potential contaminating infected cells): macrophages, granulocytes, CD8^+^ T lymphocytes, CD4^+^ T lymphocytes, and B lymphocytes. CD31 positive cells were enriched from total lung cells and then finally depleted of cells stained with the PE cocktail. (B) Flow cytometric analysis of lung cell separation following *in vivo* infection was performed on total lung cells and PE depleted/CD31+ enriched cells. Left and middle panels show representative data (1×10^6^ cells per stain) from an infected mouse (Fig. 8D). Gates were set on unstained cells, and data are representative of the four mice analyzed in Fig. 8D. Splenocytes (right panel) were included as a staining control.
(0.20 MB TIF)
