# Supplementary material for: Endothelial Cells Support Persistent Gammaherpesvirus 68 Infection
Source: PLoS Pathog. 2008 Sep 12;4(9):e1000152. doi: 10.1371/journal.ppat.1000152 (PMC2526176; doi:10.1371/journal.ppat.1000152)
Supplement: Text S1 — (0.03 MB DOC) [file ppat.1000152.s001.doc]

**Text S1.**

Cells isolated from C57/BL6 lungs were identified as endothelial cells by the following criteria:

1. Following isolation, primary lung endothelial cells grew in clusters and put out fibroblast-like extensions (Fig S4A, top panel), These cells formed monolayers within 10-14 days, showed contact inhibition, and morphology characteristic of previously published reports [78] (Fig S4A bottom panel ).
2. Endothelial cell cultures took up fluorescently labeled low density lipoprotein (LDL), as determined by fluorescent microscopy (data not shown). Uptake was comparable to that of MB114 endothelial cell lines. 3T12 fibroblasts, which do not express LDL receptors, did not take up fluorescent LDL and thus served as a negative control.
3. CD31 (PECAM-1) and CD54 (ICAM-1) are endothelial cells specific markers frequently utilized in the isolation and identification of endothelial cells [71]. CD80 and CD86 are costimulatory molecules on professional antigen presenting cells such as activated B cells, macrophages, dendritic cells, and Langerhans cells of the skin [79-81]. Following one passage, cells were positive for the endothelial cell markers CD31 and CD54 as determined by flow cytometry (Fig S4B, right panel). Primary cells were negative for CD86 with low expression of CD80, an outcome previously reported in murine lung endothelial cells isolated by similar methods [71]. CD3 lung endothelial cell lines were positive for CD31 and CD54, while 3T3 fibroblasts were negative. Both cell lines were negative for CD80 and CD86.
