# Supplementary material for: Endothelial Cells Support Persistent Gammaherpesvirus 68 Infection
Source: PLoS Pathog. 2008 Sep 12;4(9):e1000152. doi: 10.1371/journal.ppat.1000152 (PMC2526176; doi:10.1371/journal.ppat.1000152)
Supplement: Protocol S1 — Isolation and characterization of endothelial cells from murine lung tissue. (0.03 MB DOC) [file ppat.1000152.s002.doc]

**Protocol S1. Isolation and characterization of endothelial cells from murine lung tissue.**

Primary lung endothelial cells were isolated using previously published methods [71]. Briefly, C57/BL6 murine lungs were dissected into 2mm^3^ tissue blocks and washed twice in cold PBS (one minute, 210 × g). Tissue blocks were incubated in 2.5 mg/mL Dispase II, 1 mg/mL Collagenase H (Roche, Mannheim, Germany), 37°C, one hour, pipet mixing every 15 minutes. Digested tissue was then passed through a cell strainer, washed twice in cold PBS supplemented with 2.5% FBS (one minute, 210 × g), and the resultant cell suspension incubated in 25% 24G2 [74], 30 minutes, at 4°C. Following two washes in PBS supplemented with 2.5% FBS (one minute, 210 × g), cells were incubated in anti-CD31-biotin (PECAM-1, clone MEC 13.3, rat IgG2a κ, catalog number 553371) and anti-CD54-biotin (ICAM-1, clone 3E2, hamster IgG, group 1, κ, catalog number 553251) (BD Bioscience) for 30 minutes at 4°C. Following two washes in PBS supplemented with 0.5% FBS, cells were incubated with Anti-Biotin Multisort beads and separated with OctoMACS separation unit and magnet according to manufacturer’s instructions (Miltenyi Biotec Inc., Auburn, CA). Cells were resuspended at 1 × 10^5^ cells/mL in DMEM supplemented with 20% FBS, 2mM L-glutamine, 10 U/mL penicillin, and 10 μg/mL streptomycin sulfate, 1mM sodium pyruvate, 1% minimal essential amino acids (Gibco), 0.05 μM β-mercaptoethanol, 250 ng/mL fungizone, and 150 μg/mL endothelial cell growth supplement (Sigma, Saint Louis, MO), seeded in flasks coated with 2% gelatin, and incubated at 37°C with 5% CO_2_. After overnight incubation, non-adherent cells were removed and media replenished.

Cells were analyzed by flow cytometry for surface marker expression as described in Materials and Methods. The following antibodies were used: anti-CD31-biotin (PECAM-1, clone MEC 13.3, rat IgG2a κ, catalog number 553371), anti-CD54-biotin (ICAM-1, clone 3E2, hamster IgG, group 1, κ, catalog number 553251), anti-CD80-FITC (B7ּ1, clone 16-10A1, Armenian Hamster IgG2 κ, catalog number 553768), and anti-CD86-PE (B7ּ2, clone GL1, Rat IgG2a κ, catalog number 553692). CD3 lung endothelial cell lines and 3T3 fibroblasts were used as endothelial cell marker controls, positive and negative, respectively.

For analysis of uptake of low density lipoprotein (LDL) (data not shown), following incubation with 10 μg/mL DiI Ac LDL (Molecular Probes, Eugene, Oregon) cells monolayers were rinsed three times with PBS and analyzed by fluorescent microscopy. Uptake of the labeled compound was comparable to the MB114 endothelial cell line, whereas 3T12 fibroblasts did not take up the compound.
