# Supplementary figures and images for: Endothelial Cells Support Persistent Gammaherpesvirus 68 Infection
Source: PLoS Pathog. 2008 Sep 12;4(9):e1000152. doi: 10.1371/journal.ppat.1000152 (PMC2526176; doi:10.1371/journal.ppat.1000152)

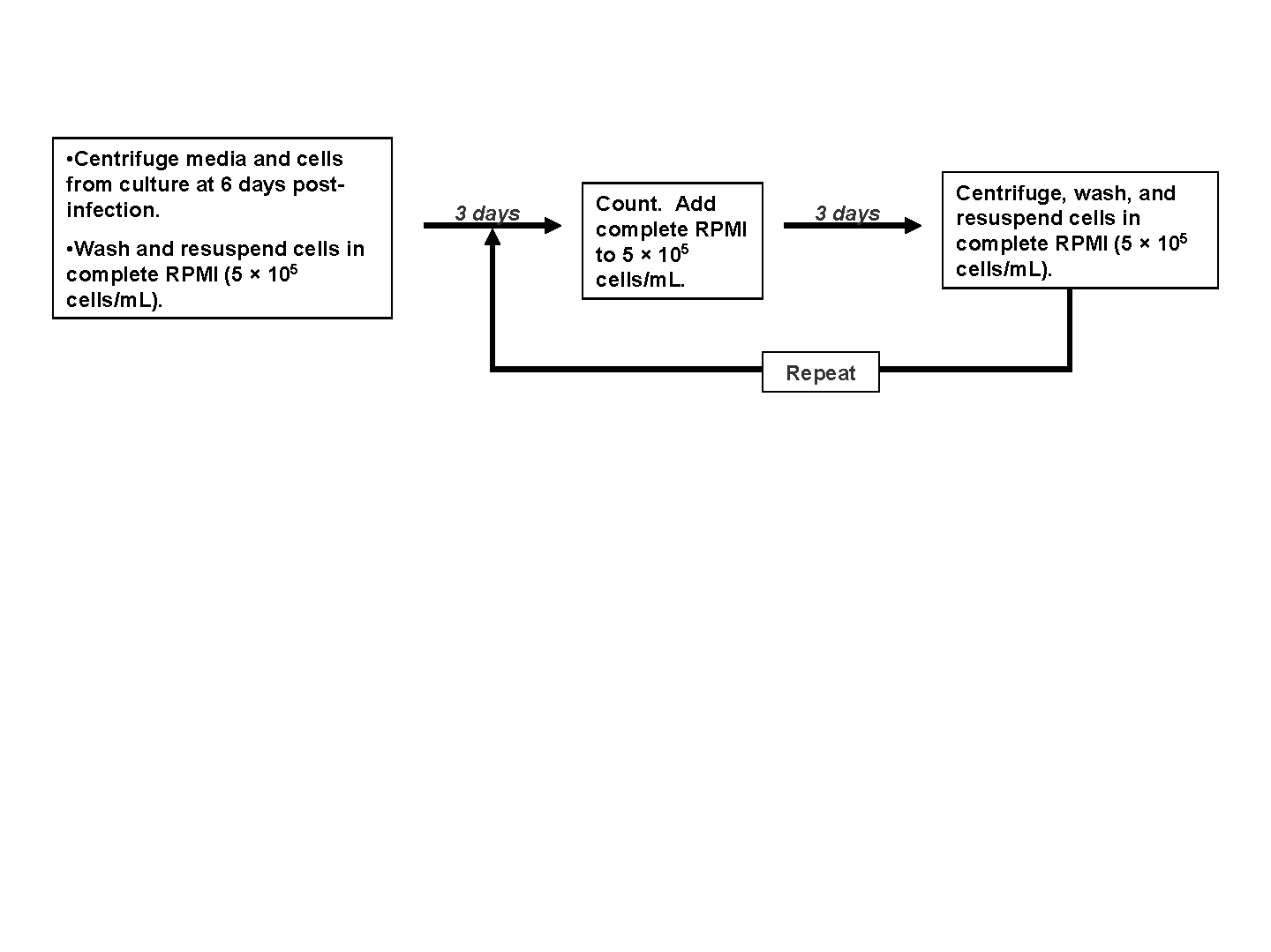

Supplement: Figure S1 — Schematic of conditions for culturing non-adherent cells following γHV68 infection. Non-adherent cells collected at six days post-infection, MOI = 5PFU/cell were cultured as described in Materials and Methods. (0.04 MB TIF) [file ppat.1000152.s003.tif]

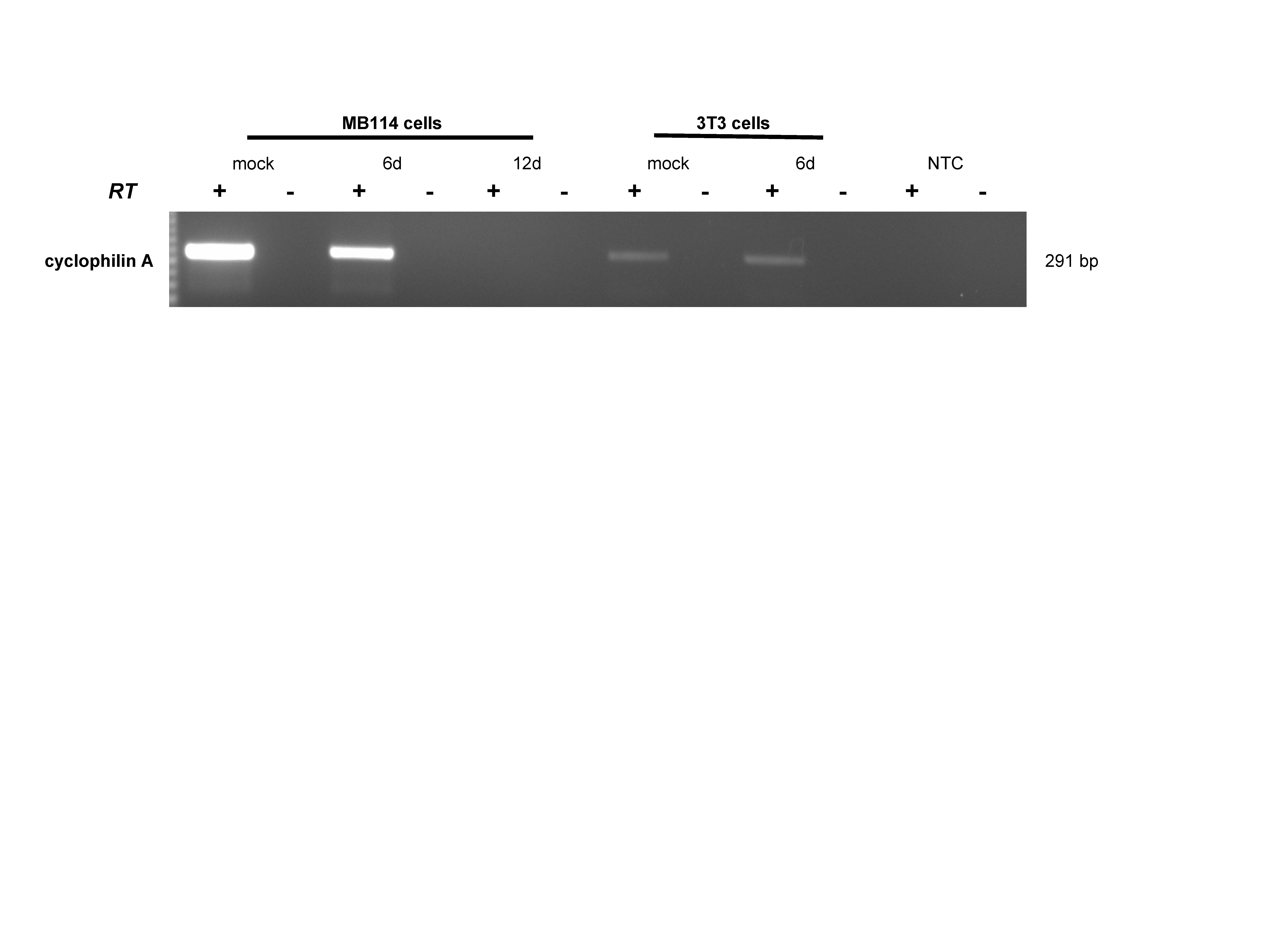

Supplement: Figure S2 — RT-PCR analysis of cyclophilin A transcript in infected cells. 100 ng of total RNA from mock infected and infected MB114 and 3T3 cells was added to each RT reaction along with primers specific for the cellular transcript cyclophilin A. No RT and no template controls are indicated. (1.88 MB TIF) [file ppat.1000152.s004.tif]

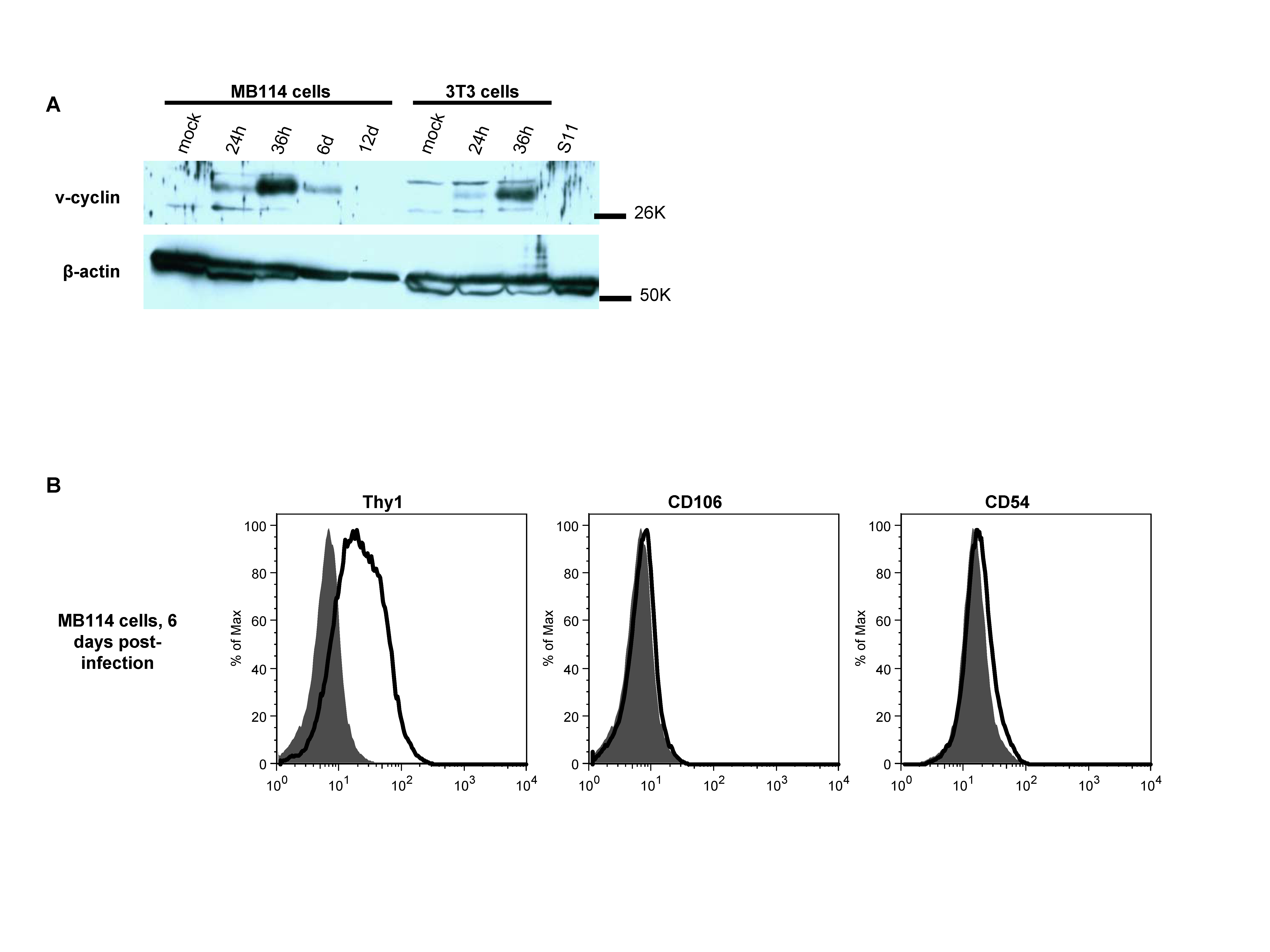

Supplement: Figure S3 — Cellular and viral protein expression following γHV68 infection. (A) MB114 cells contained viral proteins as far as 12 days post-infection. Immunoblot of γHV68 v-cyclin protein expression. 20 µg of total protein from mock infected and infected MB114 and 3T3 cells and from S11 cells were loaded per lane and blots probed with antibodies to γHV68 v-cyclin (top) and mouse β-actin (bottom). Mock infected cells were collected at 24 hours. Latent S11 cells do not express lytic viral proteins and served as a negative control. (B) The viral cyclin is not required for surface protein expression changes on infected endothelial cells. MB114 cells harvested at six days post-infection with v-cyclin.STOP γHV68 were analyzed for cell surface expression of Thy1, ICAM-1, and VCAM-1 by flow cytometry. Fluorescence was determined relative to unstained cells (grey). Results are representative from two independent experiments. (2.98 MB TIF) [file ppat.1000152.s005.tif]

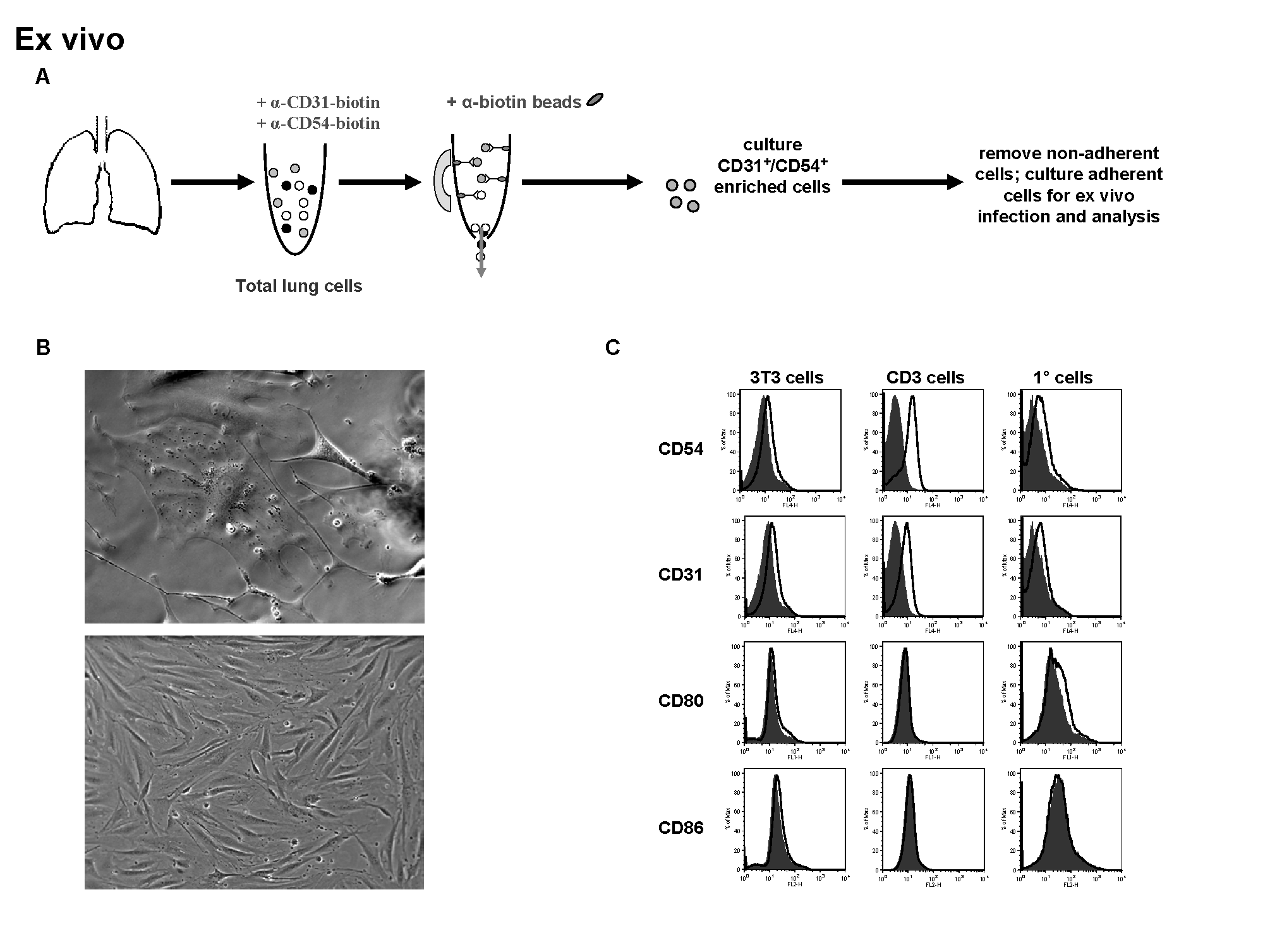

Supplement: Figure S4 — Isolation and characterization of primary murine lung endothelial cells for ex vivo culture. (A) Primary murine lung endothelial cells were isolated for ex vivo studies as described in Protocol S1. (B) Light micrographs of primary lung endothelial cells following isolation (top panel, 40X) and at confluence (bottom panel, 10X). (C) Analysis of cell surface protein expression in 3T3 fibroblast cell lines (negative control, left panel), CD3 lung endothelial cell lines (positive control, middle), and primary lung cells (right panel) by flow cytometry. Endothelial cell specific markers included CD31 and CD54. CD80 and CD86 were included as non-endothelial cell specific markers, though a previous report has identified low level CD80 expression on primary murine lung endothelial cells [71]. Fluorescence was determined relative to unstained cells (grey). Cell morphology and surface expression was similar to previously characterized primary endothelial cells [71]. (0.89 MB TIF) [file ppat.1000152.s006.tif]

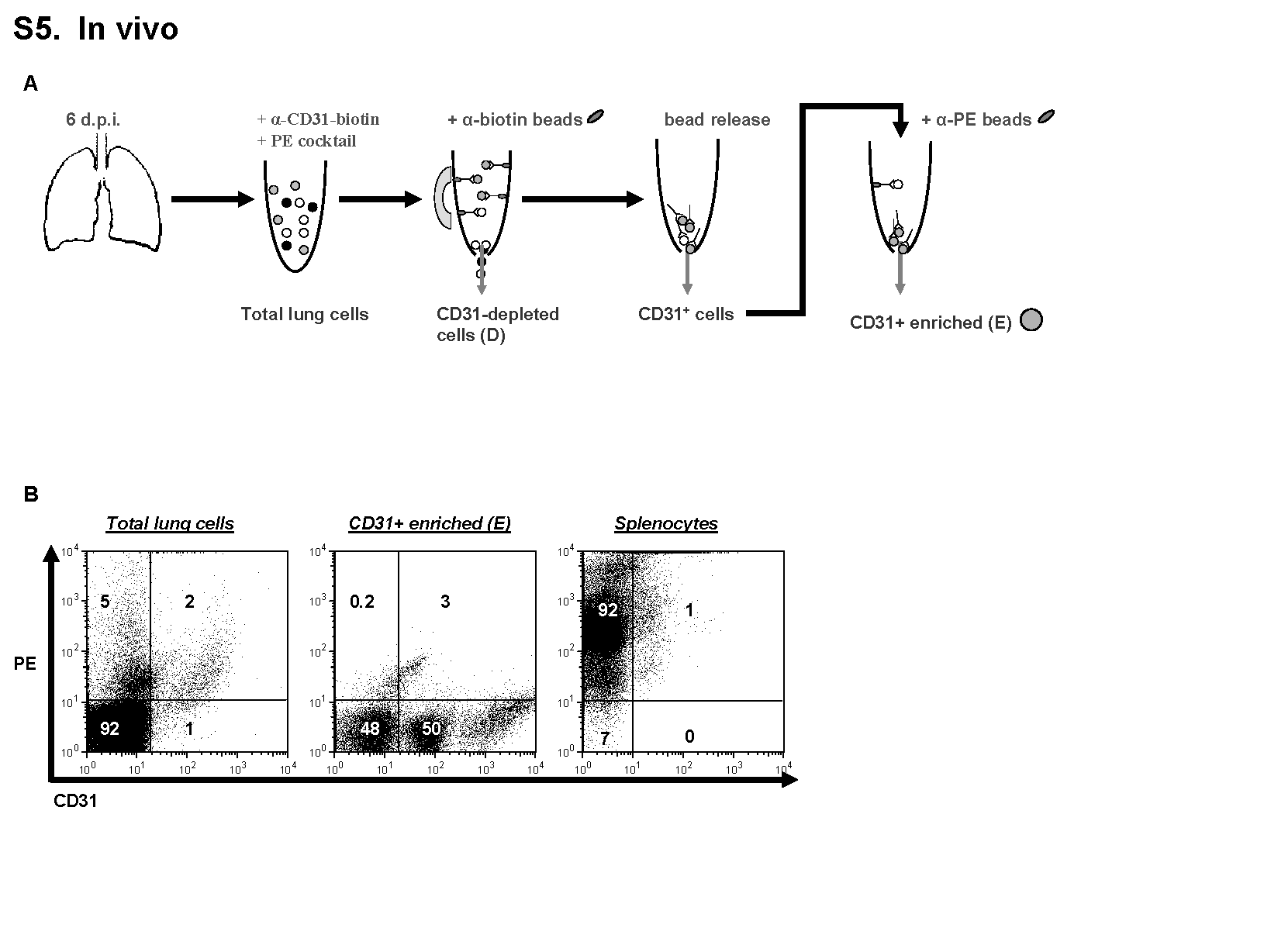

Supplement: Figure S5 — Isolation and characterization of murine lung endothelial cells infected in vivo. (A) Primary murine lung endothelial cells were isolated for analysis of infection in vivo as described in Materials and Methods. Lung cells were stained for the endothelial cell marker CD31, as well as with a cocktail of PE labeleled antibodies specific to the following cell types (which includes potential contaminating infected cells): macrophages, granulocytes, CD8+ T lymphocytes, CD4+ T lymphocytes, and B lymphocytes. CD31 positive cells were enriched from total lung cells and then finally depleted of cells stained with the PE cocktail. (B) Flow cytometric analysis of lung cell separation following in vivo infection was performed on total lung cells and PE depleted/CD31+ enriched cells. Left and middle panels show representative data (1×10^6^ cells per stain) from an infected mouse (Fig. 8D). Gates were set on unstained cells, and data are representative of the four mice analyzed in Fig. 8D. Splenocytes (right panel) were included as a staining control. (0.20 MB TIF) [file ppat.1000152.s007.tif]
